# Combined Treatment with Minocycline and an mGluR5 Antagonist Alters Resting EEG Spectral Power, but Not Sound-Evoked Responses, in a Mouse Model of Fragile X Syndrome

**DOI:** 10.1080/17590914.2025.2564628

**Published:** 2025-10-16

**Authors:** M. H. Kassir, J. W. Lovelace, D. K. Binder, I. E. Ethell, K. A. Razak

**Affiliations:** a Graduate Neuroscience Program, University of California, Riverside, California, USA; b Department of Psychology, University of California, Riverside, California, USA; c Department of Neurobiology, University of California, San Diego, California, USA; d Biomedical Sciences, University of California, Riverside, California, USA

**Keywords:** Auditory hypersensitivity, autism spectrum disorders, cortical processing, EEG phenotypes, Fragile X Syndrome, glutamate receptors, matrix metalloproteinase

## Abstract

Fragile X Syndrome (FXS) is a leading genetic cause of intellectual disability and autism-like behaviors. Glutamatergic mGluR5 receptors and matrix metalloproteinase-9 (MMP-9) are therapeutic targets to treat FXS, but clinical trials targeting each of these pathways have not been successful. Here, we tested if the electroencephalography (EEG) phenotypes associated with FXS are reversed with a novel combination of treatments affecting the two pathways. *Fmr1* knockout (KO) mice were given 10 days of CTEP (mGluR5 antagonist) alone or in combination with minocycline (MMP-9 inhibitor). EEG was recorded during resting (no acoustic stimulation) and during sound presentations (to produce sound-evoked EEG) at 1 day and 10 days after the beginning of treatment administration to test acute effects and potential tachyphylaxis. In pre-treatment WT and KO mice comparisons, we replicated previously published *Fmr1* KO mouse EEG phenotypes including elevated power in the resting gamma band, elevated single trial power, and reduced phase-locking to spectrotemporally dynamic auditory stimuli. We found that CTEP treatment alone did not show any benefit compared to vehicle in *Fmr1* KO mice after either 1 or 10 days of treatment. CTEP + minocycline reduced resting gamma band power in the *Fmr1* KO mice to a greater extent than vehicle at both treatment time points. There were no effects on sound-evoked responses. These data suggest that combined CTEP and minocycline treatment alters resting EEG measures while each treatment administered separately does not yield similar changes. High power in broadband gamma frequency correlates with irritability, stereotyped behaviors, and hyperactivity in FXS patients, suggesting a combination of drugs that reduce mGluR5 and MMP-9 activity may be beneficial in FXS.

## Introduction

Fragile X Syndrome (FXS) is a leading monogenic cause of developmental intellectual disability, affecting 1 in 4,000 males and 1 in 8,000 females (Jin & Warren, 2000; Warren & Nelson, 1994). FXS symptoms include learning, speech, and language deficits; anxiety and hyperactive behavior (Warren & Nelson, 1994); and sensory hypersensitivity (Rais et al., 2018). FXS is the leading known genetic cause of autism spectrum disorder (ASD)-like behaviors (Crawford et al., 2001). FXS is caused by a mutated Fragile X messenger ribonucleoprotein 1 gene (*Fmr1*) on the X chromosome that is preceded by repeats of CGG trinucleotide segments. An extensive number of CGG repeats (>200 copies) is present in individuals with FXS, preventing expression of Fragile X messenger ribonucleoprotein (FMRP; Kremer et al., 1991; Yu et al., 1991; Verkerk et al., 1991; Oberlé et al., 1991). FMRP regulates mRNA translation. With the loss of FMRP, there is abnormal protein synthesis and synapse development, altered excitatory to inhibitory balance, increased neuronal activity, and sensory deficits that may underlie FXS symptoms (Rais et al., 2018).

Sensory hypersensitivity is one of the most debilitating and consistent clinical symptoms in humans with FXS. Sensory hypersensitivity and underlying mechanisms can be probed objectively and across species with electroencephalography (EEG) recordings. EEG can be obtained using similar methods, stimulus paradigms, and analytical pipelines across species, including rodents and humans. Thus, EEG outcomes are a translationally relevant probe that can be used to identify parallel outcome measures and test therapeutics. Computational approaches have been developed to relate EEG abnormalities to underlying cortical circuit dysfunction (Kohl et al., 2022; Neymotin et al., 2020). In human studies, EEG outcomes are correlated with a number of clinical measures suggesting functional relevance. Therefore, EEG can be highly valuable in research on neurodevelopmental disorders to identify objective biomarkers and, furthermore, to probe circuit pathophysiology. Indeed, EEG studies strongly suggest that cortical hyper-excitability may lead to sensory hypersensitivity in FXS (Razak et al., 2021). Ethridge et al. (2019) revealed impaired habituation of the event-related potential (ERP) in response to noise bursts in adolescents and adults with FXS compared to typically-developing adults. There is elevated single trial power (STP) and increased resting EEG power in the gamma band, suggesting elevated background neural activity. In addition, compared to neurotypical participants, FXS participants have reduced phase-locking fidelity to temporally dynamic stimuli, particularly in gamma frequencies (Ethridge et al., 2019). Furthermore, high gamma power is present concurrently with reduced phase-locking to amplitude modulation of sounds at gamma frequencies, suggesting a low signal-to-noise ratio of cortical activity in the gamma range (Ethridge et al., 2019). These robust and repeatable EEG measures are correlated with clinical measures indicating relevance to daily functioning in humans with FXS. Clinical measures testing verbal and non-verbal ASD-like symptomology, such as the Social Communication Questionnaire (SCQ) scores and the ABC (which explore social communication and stereotyped behaviors), correlate poorer performance with reduced habituation of the ERP and reduced phase-locking to chirp stimulus in FXS patients (Ethridge et al., 2019), although increased gamma power in the frontal cortex (FC) of boys with FXS has been shown to correlate with better language abilities (Wilkinson & Nelson, 2021).

Similar to humans with FXS, the *Fmr1* knockout (KO) mouse model of FXS shows cortical hyperexcitability and sensory hypersensitivity (Lovelace et al., 2016, 2018; Razak et al., 2021). Electrophysiological recordings from *Fmr1* KO mice using similar paradigms as those used in humans show remarkably conserved phenotypes, including elevated gamma band power, reduced phase-locking to temporally modulated stimuli, reduced habituation to repeated stimuli, and increased response magnitudes and total power (Lovelace et al., 2016, 2018; Jonak et al., 2018, 2024; Wen et al., 2019; Croom et al., 2023; reviewed in Razak et al., 2021). Single unit recordings from the auditory cortex (AC) show increased response to auditory stimulation and increased variability of responses across trials (Rotschafer & Razak, 2013; Wen et al., 2018). Thus, enhanced background gamma oscillations (‘network noise’) may contribute to hypersensitivity, interfere with stimulus-evoked synchronization in FXS, and underlie processing deficits and increased variability to temporally modulated stimuli (Bhaskaran et al., 2023; Rotschafer & Razak, 2013; Wen et al., 2018). The similar EEG phenotypes across species provide objective and translationally-relevant outcome measures to test treatments in FXS.

There are several cellular pathways affected by the loss of FMRP in FXS. Of these, the impact of elevated metabotropic glutamatergic receptor subunit 5 (mGluR5) and matrix metalloproteinase-9 (MMP-9) pathways have received much attention. mGluR5 receptors are group I metabotropic glutamate receptors (mGluRs), which are localized to dendritic spines (Luján et al., 1997) and often act post-synaptically to regulate synaptic function and neuronal excitation (Niswender & Conn, 2010). Lack of FMRP expression results in unregulated protein synthesis downstream of mGluR5 (Dölen & Bear, 2008). mGluR5 is highly expressed in the forebrain (Bhakar et al., 2012), i.e., cortex, hippocampus, and ventral striatum (Romano et al., 1995). Therefore, changes associated with the over-activation of mGluR5 signaling may be measured using cortical EEG to understand mechanisms of sensory abnormalities in FXS. The mGluR5 pathway has been targeted for treatment of FXS and has shown promise in preclinical studies (Dölen et al., 2007; Michalon et al., 2012; Yan et al., 2005), but not in clinical trials (Berry-Kravis et al., 2018). This lack of translational success may be in part due to the choice of outcome measures across species, and it is critical to test potential treatments on objectively similar phenotypes in rodent models and in humans, such as EEG outcomes. However, very little is known about whether EEG phenotypes are affected by mGluR5 modulation. A recent study addressed this issue and showed that a single dose of CTEP, a negative allosteric modulator of mGluR5, reduced evoked potential amplitude but did not alleviate resting gamma abnormalities (Janz et al., 2025). It is not known if repeated administration of the drug has a more potent effect, or if tachyphylaxis may affect EEG measures. Furthermore, it is unknown whether targeting multiple pathways along with mGluR5 can provide more comprehensive treatment of FXS.

MMP-9 is an endopeptidase that cleaves extracellular matrix (Ethell & Ethell, 2007) and its translation is negatively regulated by FMRP (Janusz et al., 2013). In humans with FXS and in *Fmr1* KO mice, MMP-9 is elevated in multiple brain regions (Gkogkas et al., 2014; Kokash et al., 2019; Sidhu et al., 2014; Wen et al., 2018), and it is likely that this elevation disrupts various brain functions in FXS. Genetic reduction of MMP-9 in *Fmr1* KO mice reverses molecular, physiological, and behavioral deficits associated with FXS (Lovelace et al., 2016; Sidhu et al., 2014; Toledo et al., 2019; Wen et al., 2018). Similarly, pharmacological approaches to inhibit MMP-9 function with a non-specific inhibitor such as minocycline or more specific inhibitors such as SB-3CT show improvements of physiological and behavioral deficits in *Fmr1* KO mice (Lovelace et al., 2020; Pirbhoy et al., 2020; Rotschafer et al., 2012; Toledo et al., 2019). In humans as well, minocycline administration reduced deficits in sound-evoked response habituation in FXS (Schneider et al., 2013). However, in a separate study, no effects of single-dose administration of minocycline were found in humans with FXS (Erickson et al., 2025).

The translation of MMP-9 is promoted by a protein cascade downstream of mGluR5 activation (Gkogkas et al., 2014; Stoppel et al., 2017) indicating that the two pathways interact. mGluR5 activates Ras which in turn activates extracellular signal-regulated kinase (ERK), in turn activating mitogen-activated protein kinase interacting kinases (Mnk; Stoppel et al., 2017). Specifically, Mnk1 and Mnk2 phosphorylate eukaryotic Initiation Factor 4E (eIF4E), which regulates translation of MMP-9 mRNA (Stoppel et al., 2017). Pharmacological and genetic manipulations of MMP-9 activity via reduction of eIF4E phosphorylation (Gkogkas et al., 2014) or via Het models (Gkogkas et al., 2014; Lovelace et al., 2016) in the *Fmr1* KO mouse improved spine morphology, cortical habituation of responses to noise bursts, and preference for social novelty and hyperexcitable behavior. Additionally, mGluR-mediated LTD in hippocampal slices, which is normally upregulated in the *Fmr1* KO mouse model compared to wild-type (WT), serves as a robust indication of synaptic dysregulation in the absence of FMRP (Huber et al., 2002). Reduction of MMP-9 activity via pharmacological or genetic means targeting eIF4E phosphorylation results in WT-level mGluR-LTD, suggesting a direct connection between MMP-9 and mGluR-mediated synaptic function (Gkogkas et al., 2014). These data therefore provide a strong theoretical scaffold that indicates manipulating the cascade of proteins that connect mGluR5 activity to MMP-9 activity is an effective way to restore WT-like responses in *Fmr1* KO mice.

Therefore, we tested a novel combinatorial approach in which both the mGluR5 and the MMP-9 pathways were targeted simultaneously using CTEP and minocycline. The combination treatment was expected to affect a broader range of EEG phenotypes than a single drug alone (Chadwick et al., 2024; Champigny et al., 2021). We recorded EEG from the AC and FC of *Fmr1* KO mice before and after 1 day and 10 days of treatment. The longer treatment allows some time for potential circuit remodeling, as well as tests for any buildup of drug tolerance. The outcome measures we chose in this study were EEG phenotypes that are similar in rodents and humans. We tested if long term administration of an mGluR5 antagonist administered either alone (CTEP) or in combination with reduced MMP-9 signaling (CTEP + minocycline) can modify EEG responses. We tested the specific hypothesis that cortical EEG phenotypes would be ameliorated in *Fmr1* KO mice to a greater extent with CTEP + minocycline than with CTEP alone or vehicle.

## Materials and Methods

*Mice:* We tested male *Fmr1* KO and WT littermate mice on the C57bl6/J background strain (B6.129P2-Fmr1tm1Cgr/J, stock #003025) at postnatal age P60-P100. Mice were obtained by breeding WT male and *fmr1*^+/−^ female mice in an in-house breeding colony that originated from Jackson Laboratory (Bar Harbor, ME). Pups were weaned at P21 and group-housed (2–4 mice per cage) until surgery. All procedures were approved by the Institutional Animal Care and Use Committee at the University of California, Riverside. Mice were maintained in an AAALAC accredited facility in 12 h light/dark cycles and fed standard mouse chow. Food and water were provided *ad libitum*. Standard tail snip was performed on mice for genotyping (Transnetyx). Experimenters were blind to genotype. The study used a total of 25 KO and 39 WT littermates.

**Figure 1.**
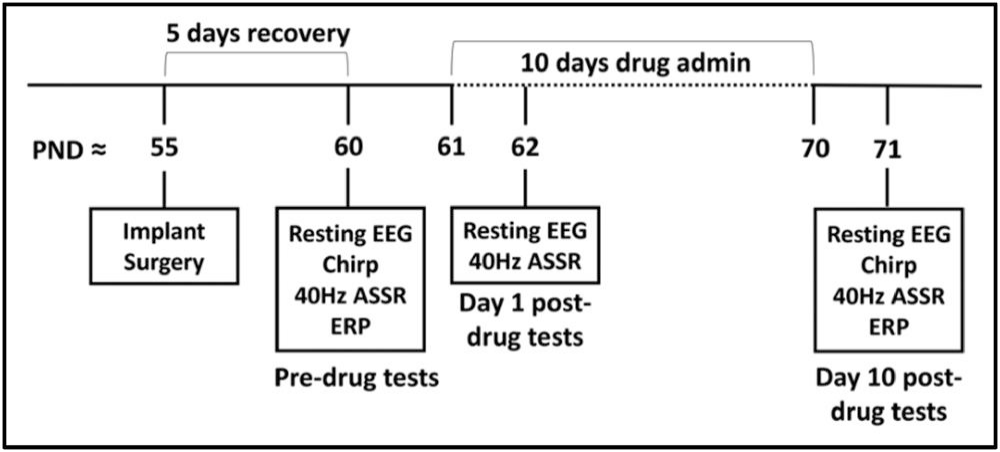
Detailed timeline of experimental procedures. PND: postnatal day; ASSR: auditory steady state responses; ERP: event related potential, which was used to generate STP: single trial power. Ages shown are for an example mouse in which pre-drug recording started at PND60. Mouse age varied from P60–100 for pre-drug tests across the study.

*Overview of Experimental Procedures* (Figure 1): All experiments were conducted on adult male C57BL/6 mice. After surgical implantation of electrodes, mice were given 4–5 full days of recovery before the start of EEG experiments. The AC and FC were recorded using epidural screw electrodes, with the occipital lobe as reference. All mice were recorded on all EEG protocols prior to drug administration onset (pre-drug). The mice then received either CTEP, CTEP + minocycline, or vehicle solution through oral gavage daily for 10 days followed by “post” EEG tests. Experiment timeline is presented in Figure 1, and sample sizes are as indicated in Table 1. All mice were genotyped after the experiment to keep experimenters blind to the genotype during surgery and procedures. Therefore, we could not pre-determine the number of WT/KO mice used. The sample in the present study had more WT than *Fmr1* KO mice. Based on variance estimation in pilot and published (e.g., Lovelace et al., 2018) EEG studies, an average of 10 (*n* = 8–12 range) mice per group are needed for 0.8 power and an alpha of 0.05. Except for one group (CTEP + minocycline-treated WT, *n* = 18), sample sizes were in that range for the present study.

**Table 1.** Sample sizes for each experimental group (genotype/treatment).

Genotypes (littermates)	Treatments	n
Fmr1-knock out (KO)	CTEP	9
	CTEP + Minocycline	8
	Vehicle	8
Wild-type (WT)	CTEP	12
	CTEP + Minocycline	18
	Vehicle	9

*EEG surgeries*: Mice were anesthetized for the entire duration of the surgery through i.p. injections of 80 mg/kg of ketamine and 10 mg/kg of xylazine. The anesthetic state of mice was monitored using the toe-pinch reflex tested every 10–15 minutes. Each mouse was placed into a standard rodent stereotaxic frame (model 930; Kopf, CA, United States). Body temperature was monitored and maintained around 37 °C using a rectal thermometer linked to a DC temperature controller system connected to a heating pad (FHC Inc). An ophthalmic ointment was administered to keep eyes moist throughout the procedure. A cosmetic hair remover (Nair) was applied on the head to clear hair and the skin was sterilized using alcohol wipes and application of betadine. An incision was made in the skin for skull exposure. Three 1 mm-diameter holes were drilled (Foredom dental drill) through the skull, exposing the right AC (−1.6 mm caudal to bregma, +4.3 mm lateral), the right FC (+3.0 mm rostral, +1.6 mm lateral) and the left occipital lobe (−4.8 mm caudal, −2.95 mm lateral). Screw electrodes (Plastics One, 00–96 X1/16) connected to a three-channel headpost (Plastics One, MS333-2-A-SPC) were implanted epidurally in the right AC and FC for EEG recording and in the left occipital lobe for reference. The screws were advanced into the skull holes until secure and to the point of contact with the dura. Dental cement was applied onto the exposed skull and around the incision site to secure the screws to the skull, sealing the surgical site completely.

*Recovery*: Antibiotic ointment was applied around the surgical site. Body temperature was monitored until mice showed signs of waking. Upon waking, mice were administered with buprenorphine, a potent analgesic (0.1 mg/kg body weight), subcutaneously. Mice were placed, singly-housed, in a new cage on top of a heat pad (Sunbeam) until fully awake and moving around. They were closely monitored for 48 hours, with buprenorphine administered subcutaneously approximately every 8 hours. Mice were allowed 4–5 days of recovery.

*Drug administration*: KO and WT littermates received one of the following treatments over the course of 10 days: CTEP (2 mg/kg per 48 hrs; Michalon et al., 2012), CTEP (1 mg/kg per 48 hrs) + minocycline (15 mg/kg per day), or vehicle (veh; 0.9% NaCl saline + 0.8% Tween-80). All treatments were delivered via oral gavage. All mice were orally gavaged daily throughout the 10-day timeline of the experiment to control for the gavage procedure across treatment regimens (Figure 1). To the groups receiving CTEP alone, CTEP was delivered once every 48 hrs, and vehicle was delivered on the days in between CTEP administration such that mice were orally gavaged daily. The groups that received CTEP + minocycline were given CTEP every 48 hrs and minocycline every 24 hrs, resulting in alternating CTEP + minocycline and only minocycline administrations across the 10 days of treatment. On the days of combination treatment, the chemicals were combined and given as a single gavage. Thus, all mice were gavaged once daily to keep this procedure consistent across groups. Administration doses and regimens were based on previous studies that tested dose and efficacy of each drug (Bilousova et al., 2009 for minocycline; Lindemann et al., 2011 for CTEP), as CTEP efficacy lasts longer than minocycline (48 hrs versus 24 hrs). We chose to use half doses of each drug in the combined treatment (1 mg/kg CTEP and 15 mg/kg minocycline) to determine if there was a synergistic effect. Control groups were administered with only vehicle solution each day during the 10-day treatment procedure.

*Drug preparation*: Drug solutions were prepared using a vehicle of 0.9% NaCl + 0.8% Tween-80, targeting an administration volume of 6 mL/kg and a target concentration of 2 mg/kg of CTEP for the CTEP treatment and 1 mg/kg of CTEP and 15 mg/kg of minocycline for the CTEP + minocycline treatment. Stock solutions of CTEP (Sigma) and minocycline (MP Biomedicals) were prepared regularly and kept frozen until time of use, when they were thawed, sonicated, vortexed, and then administered. All drugs and vehicle control solutions were administered through a stainless steel curved oral gavage tip (Cadence Science, product #7910). After each use, gavage tips were soaked in ethanol, rinsed with distilled water, and autoclaved before being used again.

*EEG:* The EEG recording and analysis methods described here followed previously published methods (Lovelace et al., 2018, 2020; Rumschlag et al., 2021). Briefly, awake and freely moving mice were placed in an arena surrounded by a custom-built Faraday cage inside a sound-insulated booth (GretchKen Industries, Oregon). They were attached to an EEG cable (Plastics One, 335-SL/3) via the implanted headpost for recording. To determine if movement caused genotype or treatment differences in power spectral density, a piezoelectric transducer was placed underneath the recording cage to detect when a mouse was moving during EEG recordings. Percent time spent moving was analyzed as a covariate in the experimental design.

Signals were recorded with filters set to high-pass (>0.5 Hz) and low-pass (<100 Hz). The gain was maintained the same (10,000×) between all recordings. Data were sampled at 2.5 kHz (Acqknowledge software) and down sampled to 1024 Hz post hoc using Analyzer 2.2 (Brain Vision Inc). TTL pulses were used to synchronize stimulus onset in each train with EEG recording. Following 10 minutes of habituation in the arena, resting EEG was recorded (5 minutes) during which no sounds were presented. Following resting EEG, ERP, chirp response, and auditory steady state response (ASSR) were recorded.

*Acoustic stimulation*: Sound stimuli were generated using RPvdsEx software and RZ6 hardware (Tucker Davis Technologies, FL, United States) and presented through a free-field speaker (MF1, Tucker-Davis Technologies, FL, United States) situated 20 cm above the floor of the arena. The speaker output was ∼70 dB SPL at the floor of the arena.

*Broadband noise stimulus* (BBN, bandwidth 1–12 kHz, 100 ms in duration, 5 ms rise/fall time) was presented at 70 dB SPL. BBN was presented in trains of 10 noise bursts at a repetition rate of 1 or 2 Hz, with a total of 100 trains per repetition rate. The interval between each stimulus train was 8 s. These stimuli were used to measure ERP and background STP across genotypes and treatments.

*Auditory steady state response* (ASSR) was recorded using clicks presented at a rate of 40 Hz. Each click train lasted 1 s in duration; there were 200 repetitions of the click train. ASSR is widely used to measure the ability of the cortex to entrain neural oscillations to a specific frequency. Measurements of power and phase-locking differences across groups are used to determine temporal processing. Robust deficits are seen in autism (Seymour et al., 2020) and schizophrenia (O’Donnell et al., 2013), suggesting the potential use as a biomarker. In addition, underlying mechanisms have been suggested for ASSR generation leading to hypotheses of circuit functional deficits (Toso et al., 2024).

*Chirp:* The chirp stimulus facilitates a rapid measurement of transient oscillatory response (delta to gamma frequency range) to auditory stimuli of varying frequencies and can be used to compare oscillatory responses in different groups in clinical and pre-clinical settings (Purcell et al., 2004). Inter-trial coherence analysis can be used to determine the ability of the neural generator to synchronize oscillations to the frequencies present in the stimulus. The chirp stimulus may be preferable over the traditional steady state stimulus in studies of children with neurodevelopmental disorders, as it can quickly and efficiently measure multiple modulation frequencies in a shorter period of time. To determine chirp responses with EEG recordings, mice were presented with a broadband noise stimulus that was amplitude-modulated with increasing frequency (1–100 Hz; upsweep chirp). Each presentation lasted 2 s in duration. There were 300 repetitions of the chirp stimulus. EEG signal acquisition was done as previously described by Lovelace et al. (2020) using the BioPac system (BIOPAC Systems, Inc).

*EEG analysis*: Resting EEG data were divided into 2 s segments, and each segment was subjected to Fast Fourier Transform (FFT) analysis using a 10% Hanning window at 0.5 Hz bin resolution. The average power density (µV^2^/Hz) was calculated for each mouse from 1 to 100 Hz. Power was binned according to spectral bands: theta (4–8 Hz), alpha (8–13 Hz), beta (13–30 Hz), low gamma (30–55 Hz), and high gamma (65–100 Hz). Chirp, ASSR, and ERP traces were processed using Morlet wavelets linearly spaced from 1 to 100 Hz using voltage (µV). Wavelet coefficients were exported as complex values for use with Inter Trial Phase Coherence (ITPC) analysis. Wavelets were run with a Morlet parameter of 10. To measure phase synchronization at each frequency across trials, ITPC was calculated as previously described (Lovelace et al., 2020). We measured baseline-corrected STP for ERP analysis. Real values of spectral power (µV^2^) were derived from Morlet wavelets. Baseline correction was done by taking the average power from −250 to −150 ms of the ERP window for each frequency layer and directly subtracting this average power from all values for their respective frequency layer in the window on a trial-by-trial basis. After baseline was corrected for each trial, the trials were averaged together.

*Statistical analysis*: Data analysis was done similarly to Lovelace et al. (2018, 2019, 2020). Briefly, statistical group comparisons of ITPC and baseline-corrected STP for the various stimulation paradigms were quantified using a Monte Carlo permutation approach. Time-frequency analysis was conducted by binning time into 256 parts and frequency into 100 parts, resulting in a 100 × 256 matrix. Non-parametric analysis was used to determine contiguous regions in the matrix that were significantly different from a distribution of 2000 randomized Monte Carlo permutations, based on previously published methods (Maris & Oostenveld, 2007). If the cluster sizes of the real genotype assignments (both positive and negative direction, resulting in a two-tailed alpha of *p* = 0.025) were larger than 97. 5% of the random group assignments, those clusters were considered significantly different between experimental conditions.

The differences between experimental conditions in resting power were analyzed using one-way MANCOVA with movement as the covariate. Specifically, movement was the proportion of time spent moving during the 5 min recording session. This was done to identify genotype effects and to control for potential effects of hyperactivity on cortical responses. Genotype and drug treatment were the independent variables, and the dependent variables were five frequency bins from theta to high gamma. This resulted in a 2 × 2 design: genotype (WT or *Fmr1* KO) × drug treatment (CTEP, CTEP + minocycline, or vehicle). Genotype or treatment comparisons were corrected for multiple comparisons using Bonferroni adjustments by using an effective α = 0.01 to account for five frequency bands.

## Results

### Relevant Experimental Group Comparisons

The following group comparisons of EEG outcomes were made to determine the effects of a treatment:

- *Fmr1* WT vs. *Fmr1* KO mice pre-treatment condition: This comparison was made to identify EEG phenotypes in KO mice before any drug administration. This can be considered a replicability and robustness experiment relative to previous work (Lovelace et al., 2018). The major difference between the studies is that Lovelace et al. (2018) used non-littermate comparisons (fully WT or *Fmr1* KO breeders), and here we use littermate comparisons. Whether the EEG phenotypes are robust to maternal genotype will be an important outcome.
- *Fmr1* KO-drug vs. WT-vehicle: This comparison was made to determine if the drug treatment produces therapeutic benefits to the *Fmr1* KO mice that make the response comparable to vehicle-treated WT mice.
- *Fmr1* KO-drug vs. *Fmr1* KO-vehicle: This comparison was made to identify specific drug (as opposed to procedural or placebo) effects on EEG of *Fmr1* KO mice. It was crucial that all drug effects in *Fmr1* KO mice were directly compared to vehicle-treated *Fmr1* KO mice. Luu et al. (2020) raised concerns about placebo effects in FXS with several clinical trials demonstrating improvements in FXS patients from placebo alone.

### EEG Phenotypes in Fmr1 KO Mice in the Pre-treatment Condition

**Figure 2.**
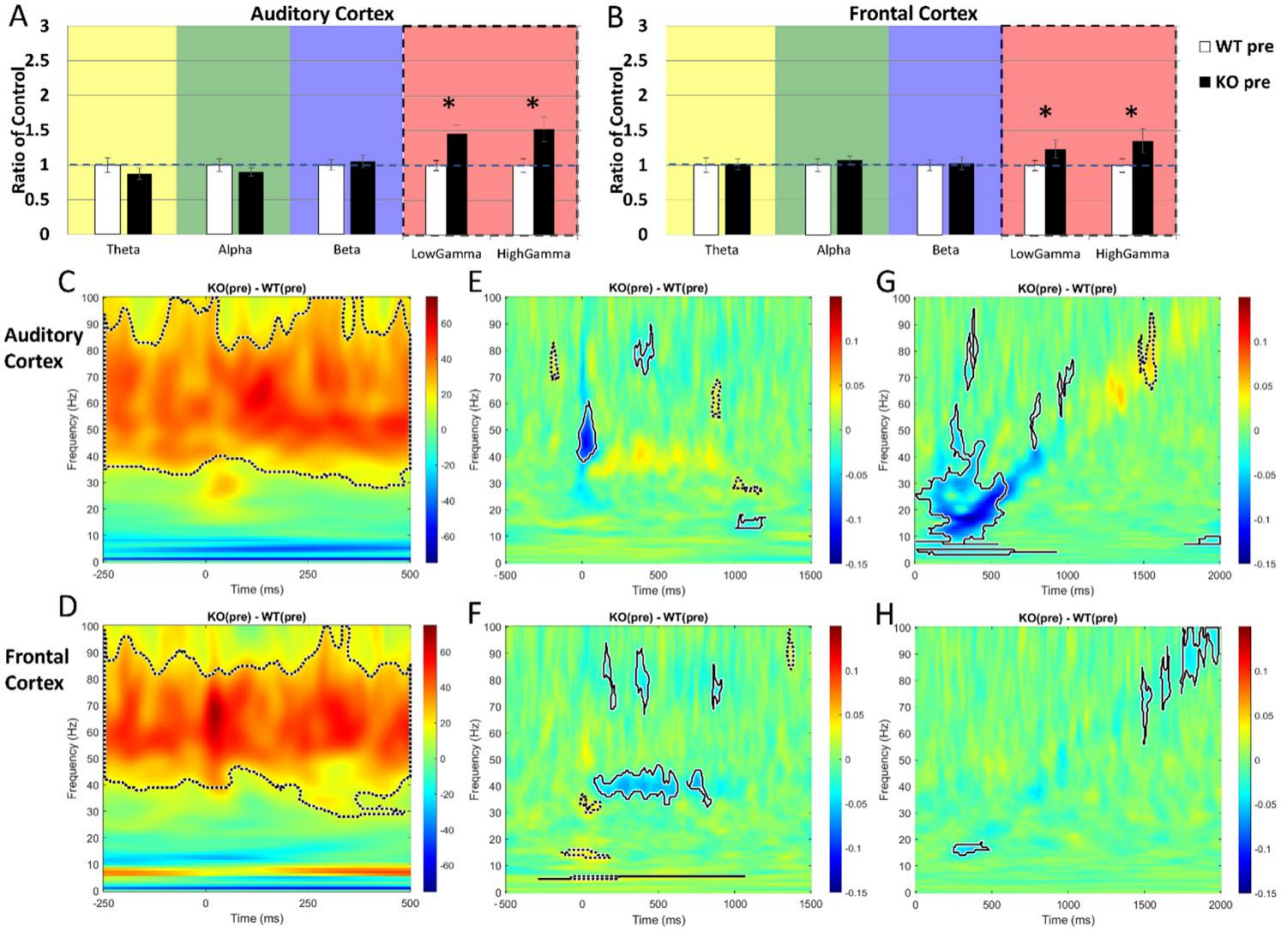
Increased resting power, elevated STP, and reduced phase-locking were observed in *Fmr1* KO mice compared to WT in the pre-drug condition. (A,B) Mean spectral power density was calculated for AC and FC prior to any drug administration per genotype. Resting EEG power in the AC (A) and FC (B) show elevated low and high gamma power compared to WT. (C,D) STP during the presentation of 100 ms noise bursts presented at a rate of 1 Hz shows elevated low and high gamma power (area inside the dotted contour is significantly different across genotypes) in the *Fmr1* KO compared to WT in the AC (C) and FC (D). (E,F) Phase-locking to the 40 Hz click train (ASSR) as measured using ITPC is reduced in AC (E) and FC (F) at 40 Hz. Each panel shows the difference in ITPC between WT and KO mice (KO-WT), with cooler colors indicating reduced phase-locking in the KO mice. Significant differences are shown as dotted line contours, with dashed lines indicating significantly higher ITPC values in the KO group compared to WT, and solid lines indicating significantly lower ITPC in the KO group compared to WT. (G,H) ITPC to the Chirp stimulus is reduced in the beta frequencies and increased in the high gamma in (G) AC and reduced in the low frequency and high gamma range in the (H) FC. These results are consistent with previously published *Fmr1* KO vs. WT EEG phenotypes recorded in the AC and FC. *n* = 25 KO and 39 WT.

We have previously reported EEG phenotypes in the *Fmr1* KO mice that are similar to those observed in humans with FXS (reviewed in Razak et al., 2021). This includes elevated resting EEG gamma band power, reduced phase-locking to spectrotemporally dynamic stimuli, and higher STP. Comparison of WT and *Fmr1* KO littermate mice used in the pre-drug condition of the present study (*n* = 25 KO and 39 WT mice) replicated the previously known EEG phenotypes (Figure 2). Figure 2(A,B) show the average spectral power density across canonical frequency bands in the AC and FC, respectively, in the pre-drug condition. The ordinate of panels in Figure 2(A,B) shows absolute power as a ratio of KO:WT mice, with ‘1’ indicating equal power, and a value greater (lower) than ‘1’ indicating higher (lower) power in the KO mice. While there was little genotype difference in the theta, alpha and beta bands, there was an elevation in the gamma band (40–100 Hz) in both cortical regions. The difference was seen in both low (40–60 Hz) and high (60–100 Hz) gamma power (AC, low gamma: *p* < .001; AC, high gamma: *p* = .004; FC, low gamma: *p* = .004; FC, high gamma: *p* = .005). MANCOVA was performed (genotype x frequency with percentage of time spent in movement as covariate) and Bonferroni correction was used for multiple comparisons (*p < 0.01). There was no difference in the percent time spent in movement during the resting EEG recording between the genotypes or treatments (Supplemental Figure 1). However, both WT and KO mice that were used in the CTEP + minocycline experiments showed relatively more movement even in the pre-treatment condition with no change in movement because of treatment. Thus, any altered movement of mice occurring alongside drug administration should not affect the treatment or genotype effects observed. Figure 2(C,D) show the difference in STP (KO - WT) in the AC and FC, respectively. Higher power in the KO mice is represented by warmer colors. The STP in each mouse was analyzed from the EEG recorded during the presentation of 100 ms noise bursts (1 Hz repetition rate; data from 2 Hz repetition rate are not shown as outcomes were similar). STP, a measure of total (background + sound-evoked) power, also showed elevated gamma (∼40–100 Hz; shown as dashed or solid line contours in the panels) power in the *Fmr1* KO mice compared to WT in both cortical regions, consistent with abnormalities in cortical circuits that generate gamma spectral power in FXS. Dotted line contours in Figure 2(C,D) delineate clusters of significant differences in the KO vs. WT comparisons: dashed lines indicate significantly higher power in the KO compared to WT, and solid lines indicate significantly lower power in the KO compared to WT.

A third robust and replicable phenotype in *Fmr1* KO mice and humans with FXS is the reduced ability of the cortex to produce consistently phase-locked responses across trials when presented with a steady state auditory stimulus such as a relatively long (1 sec) 40 Hz click train stimulus or a dynamic auditory stimulus such as the chirp. The 40 Hz ASSR is a commonly used stimulus paradigm to identify temporal processing abnormalities. The consistency of responses across trials is measured using intertrial phase coherence (ITPC). Figure 2(E,F) show that phase-locking in the 40 Hz ASSR, measured using ITPC, was reduced in *Fmr1* KO mice. The FC showed continuous reduction in the KO mice across stimulus duration, while the AC showed a reduction at the onset of the stimulus. The chirp is a sound in which a 2 sec long noise stimulus is amplitude-modulated with increasing frequency of modulation over time (upward modulated chirp). As reported previously, we observed a reduction of ITPC to the chirp in both the AC and FC (Figure 2G,H). Different bands of frequencies were affected in the AC (∼10–30 Hz) and FC (15, 60–100 Hz; shown as dashed or solid line contours in the panels), but the major effect was that ITPC in the KO mice was significantly reduced compared to WT. Taken together, we replicated the major EEG phenotypes reported in *Fmr1* KO mice that can be used to test the effects of the CTEP and CTEP + minocycline treatments.

### Effects of 1-Day CTEP Treatment on EEG Responses

The effects of CTEP treatment on EEG responses and the possibility of tachyphylaxis from continued administration of the drug were examined by recording EEG 1 day and 10 days after CTEP treatment onset in the same mice. While the full EEG data set (resting, STP, ASSR, and chirp) was collected at 10-day post treatment onset, only resting EEG and ASSR data were collected on 1-day post-treatment. The sample size for 1-day post CTEP recordings is: *n* = 9 *Fmr1* KO with CTEP (KO-CTEP), *n* = 8 *Fmr1* KO with vehicle (KO-veh), *n* = 9 WT with vehicle (WT-veh), and *n* = 12 WT with CTEP (WT-CTEP, presented in Supplemental Figures 2, 8).

**Figure 3.**
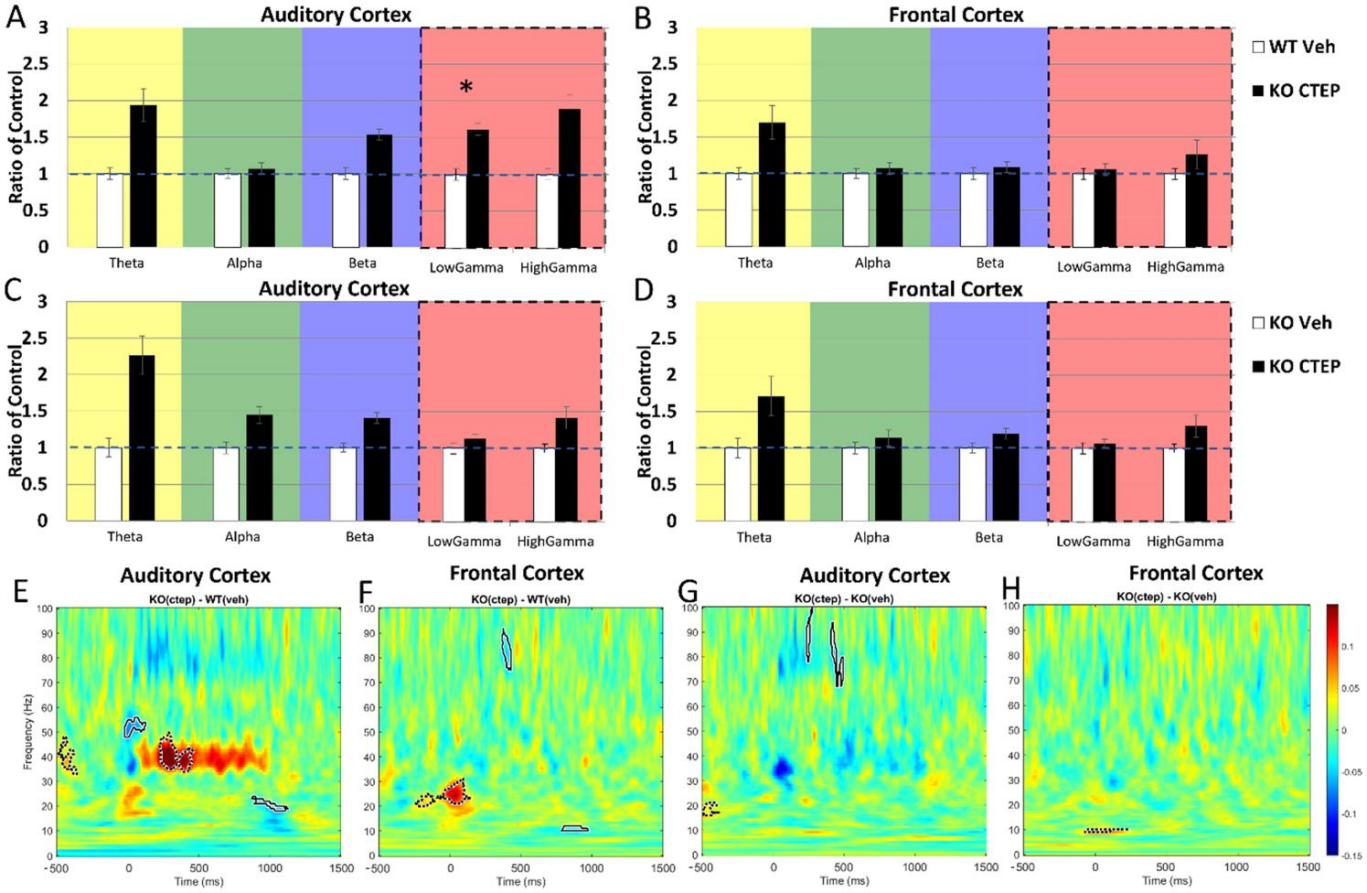
Day 1 post-treatment with CTEP resulted in similar spectral power in resting EEG between KO and WT in the FC, but not in the AC where elevated low gamma power persists. However, CTEP treatment is not significantly different from vehicle, suggesting an effect of handling, not treatment. (A–D) Mean power spectral density was calculated for AC and FC Day 1 post treatment onset. Resting EEG power in the (A) AC shows persisting elevated low gamma power in the KO treated with CTEP compared to WT treated with vehicle, while showing similar power across these groups in the (B) FC. Resting EEG power in the (C) AC and (D) FC of KO Day 1 post-CTEP onset compared to vehicle-treated KO shows no difference between drug and vehicle. (E) ITPC in response to a 40 Hz click train (ASSR) is elevated in the AC of KO mice treated with CTEP compared to WT treated with vehicle on Day 1 post treatment onset. (F) ITPC in the FC is similar between KO-CTEP and WT-veh. (G,F) ITPC in response to a 40 Hz click train (ASSR) is similar between KO-CTEP and KO-veh in the (G) AC and (H) FC. This suggests that improvements shown in E, F is an effect of handling, not treatment. *n* = 9 KO-CTEP, 9 WT-veh, 8 KO-veh.

Figure 3 shows EEG responses after 1 day of CTEP treatment. For data analysis, MANCOVA was performed (genotype or treatment x frequency band, with movement as covariate) and Bonferroni correction was made for multiple comparisons (*p < 0.01). In the AC, there was no CTEP-driven improvement of gamma power in the KO group compared to WT treated with vehicle, as low gamma power is elevated in the KO compared to WT (Figure 3A; AC, low gamma: *p* = 0.003). Resting power in the AC of *Fmr1* KO mice was similar between CTEP and vehicle (Figure 3C; all *p* > 0.01), indicating no effect of treatment. In the FC, CTEP treatment reduced gamma power to WT-veh levels (Figure 3B; all *p* > 0.01), however, because there was no difference between KO-CTEP and KO-veh (Figure 3D; all *p* > 0.01), we interpret this to indicate an effect of the experimental procedure, and not a specific drug outcome. None of the other frequency bands showed any significant effects of treatment or vehicle (all *p* > 0.01).

CTEP-treated KO mice did not show improved sound-driven phase-locking in the 40 Hz ASSR (Figure 3E–H). In the AC (Figure 3E,G), while ITPC increased in the CTEP-treated KO mice compared to vehicle-treated WT mice (Figure 3E; dashed-line cluster around 40 Hz), the treatment outcome in the KO mice was similar for drug and vehicle (Figure 3G; no clusters around 40 Hz). The outcome was essentially similar in the FC (Figure 3F,H). Taken together, these data show that 1-day CTEP treatment did not have specific effects on resting EEG power or on sound-driven phase-locking fidelity. Differences seen in resting and evoked power were likely a placebo effect, as vehicle conditions were not significantly different from drug treatment in the KO mice.

### Effects of 10 Days of CTEP Treatment on EEG Responses

**Figure 4.**
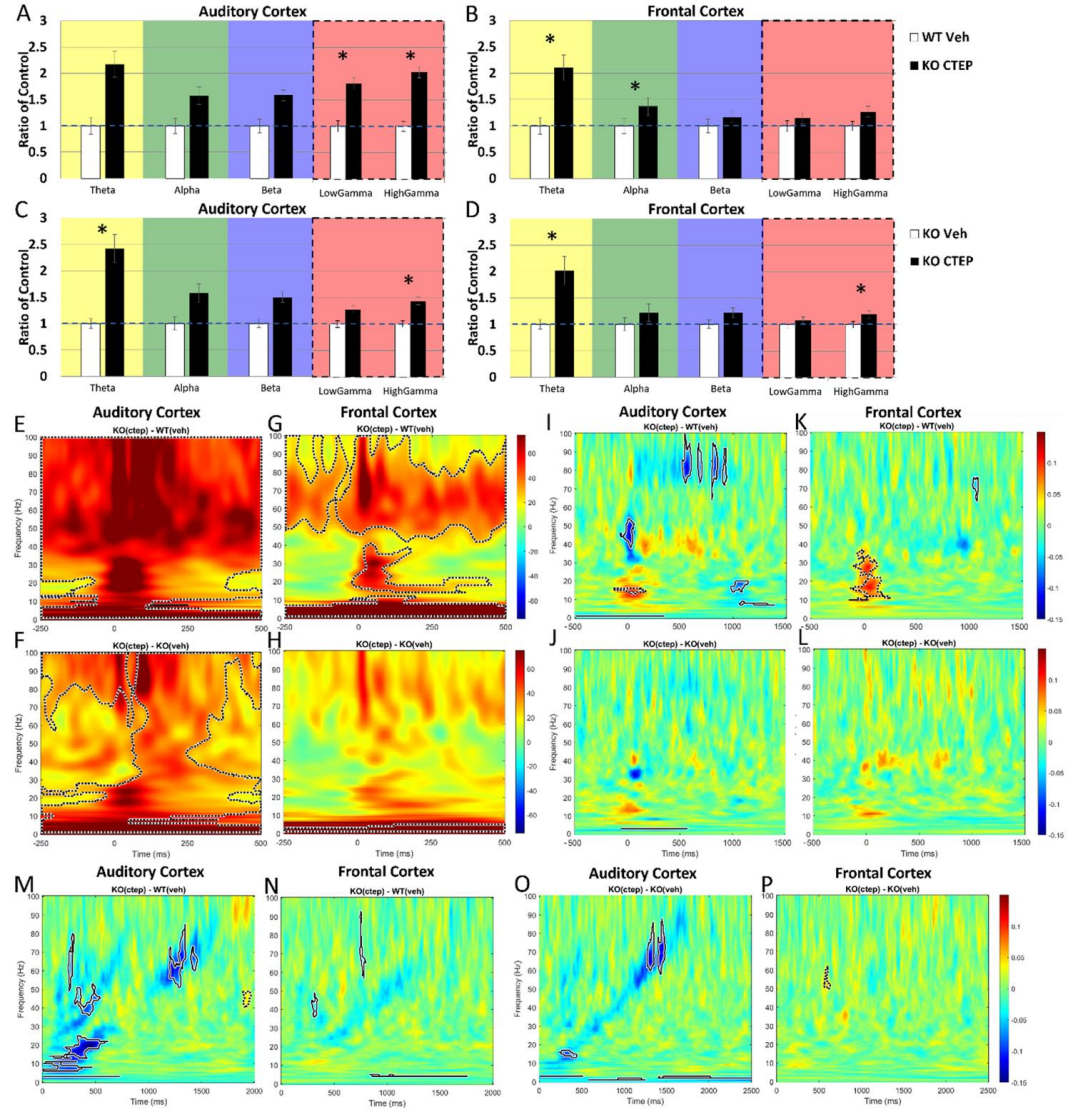
Day 10 post-treatment with CTEP displayed an exacerbated *Fmr1* KO phenotype in resting EEG and in STP responses to noise bursts in the KO compared to WT, and did not improve ITPC to amplitude-modulated signals in the KO compared to vehicle treatment. (A-D) Mean power density of AC and FC at Day 10 post treatment onset. Resting EEG power in the (A) AC of KO-CTEP compared to WT-veh shows persisting elevated low and high gamma levels in the KO. (B) There is no difference in the FC between KO and WT in the gamma band, although theta and alpha power are elevated in the KO compared to WT. (C) Comparing resting EEG power between CTEP and vehicle treatment in the KO shows elevated high gamma power in the AC of KO-CTEP compared to KO-veh, and increased power in theta. (D) These effects are also present in the FC. This suggests that Day 10 post-treatment with CTEP in the KO is worse than vehicle treatment. MANCOVA was performed (genotype or treatment x freq, movement as covariate) with Bonferroni correction for multiple comparisons: *p < 0.01. (E,G) KO-CTEP shows persisting elevated STP gamma power compared to WT-veh. STP during the presentation of 100 ms noise bursts presented at a rate of 1 Hz shows elevated power in the *Fmr1* KO treated with CTEP across all frequencies in the (E) AC and (G) FC compared to WT-veh, including in low frequencies which was not present in the pre-drug comparison (Figure 1), suggesting an exaggerated KO phenotype. (F,H) Compared to vehicle, treatment with CTEP did not reduce STP in the (H) FC and increased STP in the (F) AC, further demonstrating the enhanced KO phenotype upon CTEP treatment. (I-L) Phase-locking to the 40 Hz click train (ASSR) as measured using ITPC in the CTEP-treated KO (I,L) and in the vehicle-treated KO (J,L). ITPC in CTEP-treated KO is similar to WT-veh in the FC (K) but remains lower than WT-veh in the AC (I). (J,L) Treatment with CTEP results in similar ITPC levels as vehicle, suggesting no treatment effect. (M) ITPC to the chirp stimulus in the CTEP-treated *Fmr1* KO is shown compared to WT-veh in the AC (KO-ctep minus WT-veh). Significant clusters around the chirp indicate lower ITPC in the KO compared to WT around ∼1-20 Hz and ∼60-80 Hz amplitude modulation. (N) ITPC to the chirp stimulus in the CTEP-treated *Fmr1* KO is similar to WT-veh in the FC (no significant clusters around the chirp stimulus). (O) CTEP treatment in KO mice resulted in lower ITPC compared to vehicle treatment in the AC, with clusters around ∼15 Hz and ∼60–80 Hz of the chirp stimulus indicating lower ITPC in the KO-CTEP compared to KO-veh, demonstrating worsened ITPC with CTEP treatment in KO. (P) ITPC to the chirp stimulus in the CTEP-treated *Fmr1* KO is similar to KO-veh in the FC (no significant clusters around the chirp stimulus). *n* = 9 KO-CTEP, 9 WT-veh, 8 KO-veh.

The sample sizes for EEG recordings performed 10 days post-CTEP treatment onset were: *n* = 9 *Fmr1* KO with CTEP, *n* = 8 *Fmr1* KO with vehicle, *n* = 9 WT with vehicle, and *n* = 12 WT with CTEP (WT-CTEP data is presented in Supplemental Figures 2, 8). CTEP treatment over 10 days had no improvements on resting or sound-driven EEG responses in KO mice (Figure 4). In the AC, 10-day CTEP-treated KO mice continued to show increased gamma band power compared to WT-veh (Figure 4A; AC, low gamma: *p* < .001; AC, high gamma: *p* < 0.001) and compared to KO-veh (Figure 4C; AC, theta: *p* = 0.004; AC, high gamma: *p* < 0.001). In the FC, CTEP treatment reduced gamma power to WT-veh levels (Figure 4B; *p* > 0.01). However, CTEP-treated KO mice showed increased gamma power compared to vehicle-treated KO mice (Figure 4D; FC, high gamma: *p* = 0.003). CTEP treatment increased low-frequency power (theta, alpha) in the FC of KO mice (Figure 4B; FC, theta *p* = 0.007; FC, alpha *p* = 0.004). The elevated theta power in the KO-CTEP mice when compared to WT-veh is also present when compared to KO-veh (Figure 4D; FC, theta: *p* = 0.005), indicating an effect due to CTEP administration. MANCOVA was performed (genotype or treatment x frequency, with movement as covariate) and Bonferroni correction was made for multiple comparisons (*p < 0.01).

*Fmr1* KO mice with 10 days of CTEP treatment continued to show elevated STP during sound stimulation compared to vehicle-treated WT mice. STP during the presentation of 100 ms noise bursts showed elevated power in the *Fmr1* KO treated with CTEP across all frequencies in the AC (Figure 4E) and FC (Figure 4G) compared to WT-veh, including in low frequencies (∼1–40 Hz) which were not present in the pre-treatment comparisons (Figure 2). Compared to vehicle, treatment with CTEP in the KO mice increased STP in the AC (Figure 4F; all frequencies) and did not significantly change STP in the FC (Figure 4H; clusters were found only around 0–10 Hz). These data suggest that CTEP, compared to vehicle, exaggerated noise-burst responses in the *Fmr1* KO mice.

CTEP administration to *Fmr1* KO mice over 10 days did not improve their ITPC to ASSR stimulus or to chirp. ITPC to ASSR stimulus in CTEP-treated KO mice was similar to WT-veh in the FC (Figure 4K; no significant clusters around 40 Hz) but remained lower than WT-veh in the AC (Figure 4I; cluster indicating lower ITPC at 40 Hz in the KO compared to WT at the stimulus onset). In the AC, there was reduced ITPC in the KO-CTEP group compared to WT-veh group at the 80 Hz harmonic and higher ITPC at ∼15 Hz (Figure 4I; cluster around 80 Hz harmonic indicating lower ITPC in the KO compared to WT; small cluster around ∼15 Hz indicating higher ITPC in the KO group compared to WT). In the FC, there was increased ITPC in the KO-CTEP group compared to WT-veh at frequencies lower than 40 Hz (Figure 4K; cluster around 10–35 Hz indicating higher ITPC in the KO group compared to WT). Treatment of *Fmr1* KO mice with CTEP resulted in similar ITPC levels as vehicle treatment in both cortical regions, suggesting no treatment effect (Figure 4J,L; no significant clusters). Likewise, ITPC to the chirp stimulus in the CTEP-treated *Fmr1* KO mice remained lower than WT-veh in the AC (Figure 4M; clusters around the chirp indicate lower ITPC in the KO group compared to WT group around ∼1–20 Hz and ∼60–80 Hz amplitude modulation) but showed similar ITPC in the FC (Figure 4N; no significant clusters around the chirp stimulus). However, CTEP treatment in KO mice resulted in lower ITPC compared to vehicle treatment in the AC (Figure 4O; clusters around ∼15 Hz and ∼60–80 Hz of the chirp stimulus indicating lower ITPC in the KO-CTEP group compared to KO-veh group) and was not different in the FC (Figure 4P; no significant clusters around the chirp stimulus). Taken together, we interpret these data to mean that 1- or 10-day CTEP treatment had no normalizing effect on EEG phenotypes in the *Fmr1* KO mice.

### Effects of 1-Day Combined CTEP + Minocycline Treatment on EEG Responses

**Figure 5.**
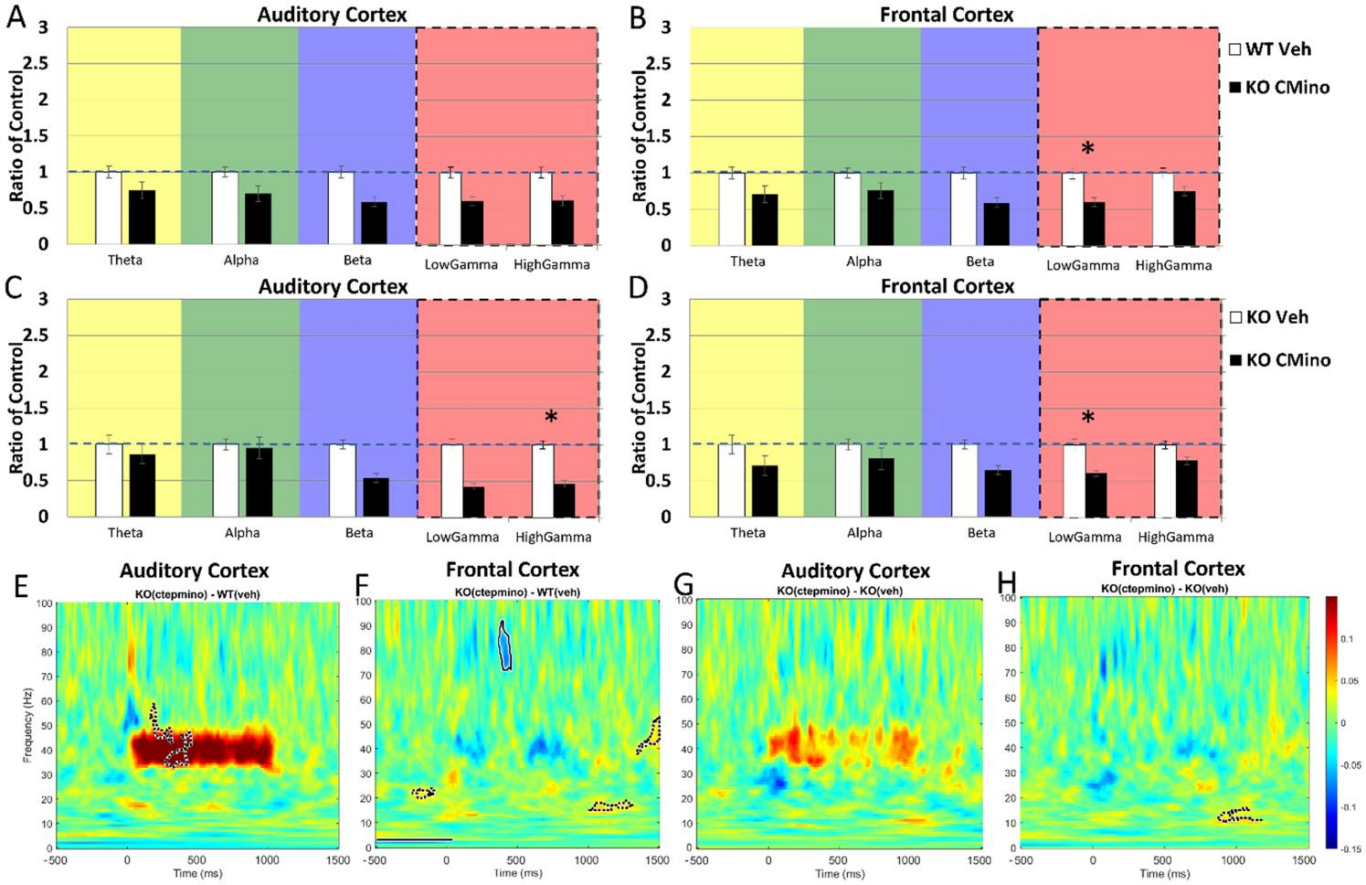
Day 1 post-treatment with CTEP + minocycline in the KO resulted in similar resting spectral power as the WT-veh in the AC and FC. Treatment with CTEP + minocycline reduced high gamma and low gamma power in the AC and FC, respectively, compared to vehicle. (A-D) Mean power density was calculated for AC and FC Day 1 post treatment onset. CTEP + minocycline results in similar EEG levels in KO compared to WT in the (A) AC and (B) FC. Resting EEG power in the (C) AC and (D) FC of KO Day 1 post-CTEP + minocycline onset compared to vehicle-treated KO shows reduced high and low gamma power, respectively. (E) ITPC in response to a 40 Hz click train (ASSR) is elevated in the AC of KO mice treated with CTEP + minocycline compared to WT treated with vehicle on Day 1 post treatment onset. (F) ITPC in the FC is similar between KO-Cmino and WT-veh. (G,F) However, ITPC in response to a 40 Hz click train (ASSR) is similar between KO-Cmino and KO-veh in the (G) AC and (H) FC, suggesting an effect of handling, not treatment. *n* = 8 KO-Cmino, 9 WT-veh, 8 KO-veh.

The sample sizes for the combined treatment were: *n* = 8 *Fmr1* KO with CTEP + minocycline (KO-Cmino), *n* = 9 WT with vehicle, *n* = 8 *Fmr1* KO with vehicle, and *n* = 18 WT with CTEP + minocycline (WT-Cmino, presented in Supplemental Figures 3, 7). At 1-day post drug administration onset of CTEP and minocycline, resting EEG gamma power was similar in the *Fmr1* KO mice compared to WT-veh in the AC (Figure 5A; *p* > 0.01). In the FC, combined CTEP and minocycline treatment resulted in a normalization of high gamma power and reduced low gamma power to below WT-veh levels (Figure 5B; FC, low gamma: *p* = 0.01; FC, high gamma: *p* > 0.01). Comparison of KO-Cmino mice with KO-veh mice showed a significant reduction in high and low gamma power in the AC and FC in the drug condition, respectively, indicating a specific effect of the combined drug treatment at these frequencies in their respective cortices (Figure 5C,D; AC, high gamma: *p* = 0.002; FC, low gamma: *p* = 0.003). MANCOVA was performed (genotype or treatment x frequency with movement as covariate) and Bonferroni correction was made for multiple comparisons (*p < 0.01).

ITPC in response to a 40 Hz ASSR was elevated in the AC of *Fmr1* KO mice treated with CTEP and minocycline compared to WT treated with vehicle at 1-day post-treatment onset (Figure 5E; dashed clusters around 40 Hz ASSR indicating higher ITPC in the KO compared to WT). ITPC in the FC was similar between KO-Cmino and WT-veh (Figure 5F; no significant clusters around 40 Hz). However, ITPC in response to a 40 Hz ASSR was similar between KO-Cmino and KO-veh in the AC (Figure 5G; no significant clusters around 40 Hz) and FC (Figure 5H; no significant clusters around 40 Hz), indicating that no specific drug effect was present on phase-locking in the ASSR following 1 day of combined treatment.

Taken together, resting EEG gamma power in the *Fmr1* KO mice was improved following 1 day of combined treatment (CTEP + minocycline). Specifically, there was normalization of high gamma power in the AC and of low gamma power in the FC. The combined treatment induced no specific change in ASSR.

### Effects of 10 Days of Combined CTEP + Minocycline Treatment on EEG Responses

**Figure 6.**
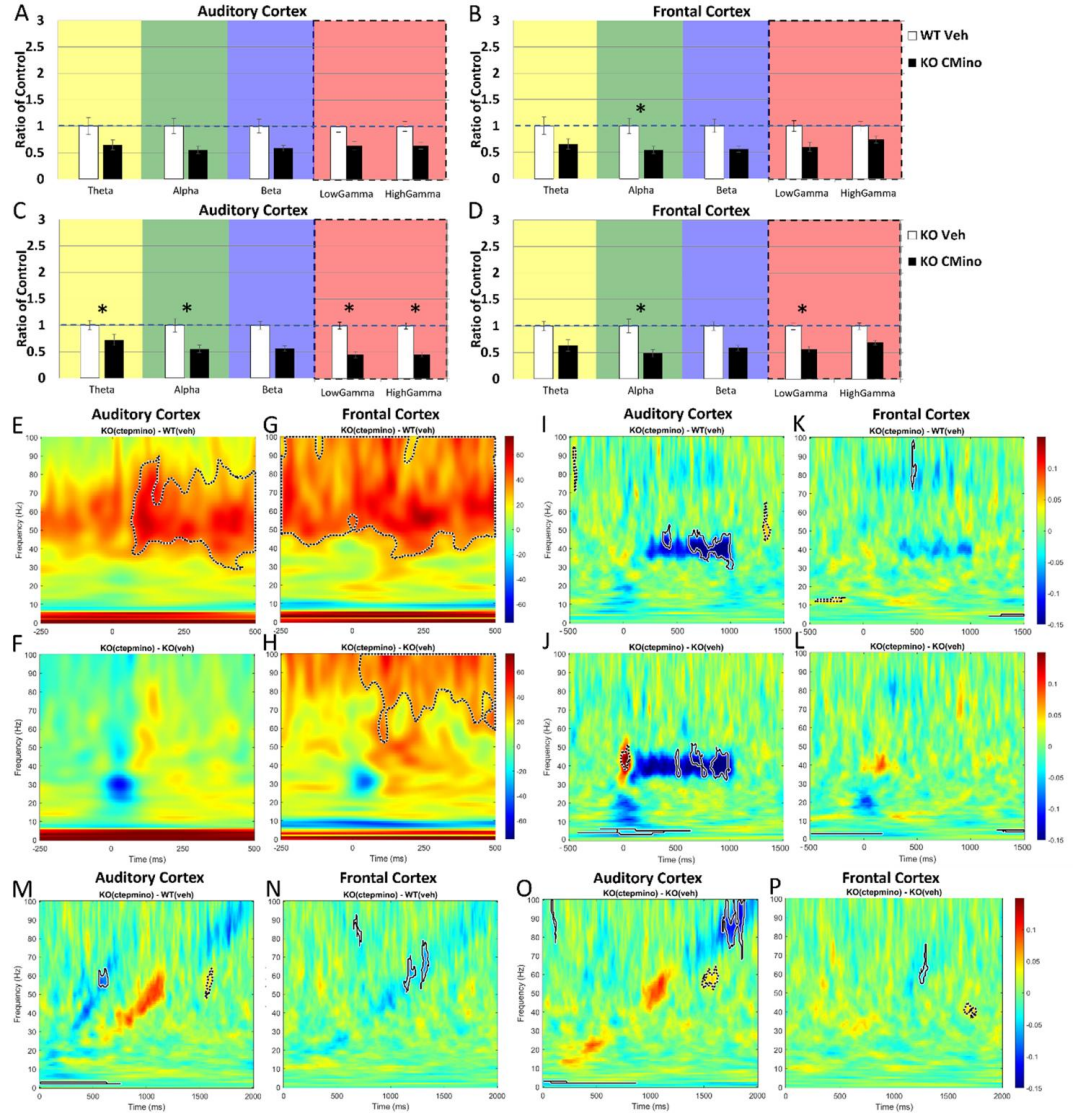
Day 10 post-treatment with CTEP + minocycline restored low and high gamma levels in the AC and FC in KO compared to WT, while not improving STP nor ITPC. (A-D) Mean power density of AC and FC at Day 10 post treatment onset. (A) KO treated with CTEP + minocycline show similar resting EEG power as WT-veh in the AC (all *p* > 0.01). (B) In the FC, KO treated with CTEP + minocycline show significantly reduced alpha band power compared to WT-veh, while theta, beta, low gamma, and high gamma bands show similar power levels to WT-veh. Notably, low and high gamma power is no longer greater in the KO group compared to WT. (C,D) Compared to vehicle, treatment with CTEP + minocycline significantly (C) lowered low and high gamma power in the AC and (D) reduced low gamma power in the FC of KO mice, suggesting a treatment effect restoring low and high gamma power to WT levels that is different from vehicle. Additionally, (C) theta and alpha power are reduced in the AC and (D) alpha power is reduced in the FC in the treatment group compared to vehicle. MANCOVA was performed (genotype or treatment x freq, movement as covariate) and Bonferroni correction for multiple comparisons: *p < 0.01. (E,G) Day 10 post treatment onset with CTEP + minocycline in the KO shows persisting elevated STP gamma power in the KO compared to WT. STP during the presentation of 100 ms noise bursts presented at a rate of 1 Hz shows elevated power in the *Fmr1* KO treated with CTEP + minocycline across gamma frequencies in the (E) AC and (G) FC compared to WT (significant clusters in the ∼40-90 Hz range in the AC and 40-100 Hz range in the FC). (F,H) Compared to vehicle, treatment with CTEP + minocycline did not reduce STP in the (F) AC (no significant clusters) and increased STP in the (H) FC (signficant clusters in the ∼60-100 Hz range). (I-L) Phase-locking to the 40 Hz click train (ASSR) as measured using ITPC in the CTEP + minocycline-treated KO (I,L) and in the vehicle-treated KO (J,L). ITPC in CTEP + minocycline-treated KO is (I) reduced compared to WT in the AC (significant clusters around 40 Hz across the stimulus presentation duration) and (K) similar to WT in the FC (no significant clusters). (J,L) Treatment with CTEP + minocycline results in (J) lower ITPC levels in the AC compared to vehicle and (L) similar ITPC levels as vehicle, suggesting no treatment effect. (M) ITPC to the chirp stimulus in the *Fmr1* KO mice treated with CTEP + minocycline was similar to WT-veh levels in the AC (no significant clusters). (N) *Fmr1* KO mice treated with CTEP + minocycline showed lower ITPC compared to WT-veh levels in the FC (significant cluster in the ∼50-70 Hz amplitude modulation range). (O,P) CTEP + minocycline treatment in KO mice resulted in lower ITPC compared to vehicle treatment in the (O) AC (cluster in the ∼70-100 Hz amplitude modulation range) and (P) FC (cluster at ∼60 Hz amplitude modulation). *n* = 8 KO-Cmino, 9 WT-veh, 8 KO-veh.

The sample size for the combined treatment measured 10 days post-treatment onset was: *n* = 8 KO with CTEP + minocycline, *n* = 9 WT with vehicle, *n* = 8 KO with vehicle, *n* = 18 WT with CTEP + minocycline (Supplemental Figures 3, 7). Like the 1-day treatment, the 10-day treatment with CTEP and minocycline restored gamma levels in the AC and FC in *Fmr1* KO mice compared to WT-veh, while not affecting STP and ITPC in the ASSR (Figure 6). *Fmr1* KO mice treated with CTEP and minocycline showed similar resting EEG gamma power in the AC (Figure 6A; *p* > 0.01) and FC (Figure 6B; *p* > 0.01) compared to WT-veh. Compared to KO-veh, CTEP + minocycline treatment in the *Fmr1* KO mice significantly lowered gamma power in the AC (Figure 6C; AC, low gamma: *p* = 0.002; AC, high gamma: *p* < 0.001) and reduced low gamma power in the FC (Figure 6D; FC, low gamma: *p* = 0.006), suggesting a specific treatment effect restoring gamma power to WT levels when administering CTEP and minocycline that is different from vehicle administration alone. Additionally, theta and alpha power were reduced in the AC (Figure 6C; AC, theta: *p* = 0.003; AC, alpha: *p* = 0.002) and alpha power was reduced in the FC (Figure 6D; FC, alpha: *p* < 0.001) in the combined treatment group compared to vehicle. MANCOVA was performed (genotype or treatment x frequency with movement as covariate) and Bonferroni correction was made for multiple comparisons (*p < 0.01).

Day 10 post-treatment onset with CTEP + minocycline in the *Fmr1* KO mice did not affect STP gamma power in the *Fmr1* KO mice compared to WT-veh (Figure 6E–H). STP during the presentation of 100 ms noise bursts showed elevated power in the *Fmr1* KO treated with CTEP and minocycline across gamma frequencies in the AC (Figure 6E; ∼40–90 Hz) and FC (Figure 6G; ∼40–100 Hz) compared to WT-veh. Treatment with CTEP + minocycline for 10 days increased STP in the gamma range in the FC of the KO-Cmino group compared to KO-veh (Figure 6H; ∼50–100 Hz), but it did not alter STP in the AC (Figure 6F; no significant clusters). Phase-locking to the 40 Hz click train (ASSR) or to the chirp stimulus was not improved in the KO-Cmino group compared to WT- or KO-veh (Figure 6I–L, M–P). ITPC to the ASSR stimulus in KO-Cmino was reduced compared to WT-veh in the AC (Figure 6I; significant cluster around 40 Hz) and similar to WT-veh in the FC (Figure 6K; no clusters around 40 Hz). In KO mice, treatment with CTEP + minocycline resulted in higher ITPC at the onset of the 40 Hz click stimulus and lower ITPC levels throughout the duration of the stimulus in the AC compared to vehicle (Figure 6J; cluster at stimulus onset indicating higher ITPC in the treatment group around 40 Hz, followed by clusters around 40 Hz indicating lower ITPC in the treatment group) and similar ITPC levels as vehicle in the FC (Figure 6L; no clusters around 40 Hz), indicating no specific treatment effect on phase-locking in the 40 Hz ASSR.

ITPC to the chirp stimulus in the *Fmr1* KO mice treated with CTEP + minocycline was similar to WT-veh levels in the AC (Figure 6M; no significant clusters) and lower in the FC (Figure 6N; cluster in the ∼50-70 Hz amplitude modulation range indicating lower ITPC in the KO group). However, CTEP + minocycline treatment in KO mice resulted in lower ITPC compared to vehicle treatment in the AC (Figure 6O; cluster in the ∼70–100 Hz amplitude modulation range indicating lower ITPC in the treatment group) and FC (Figure 6P; cluster at ∼60 Hz amplitude modulation indicating lower ITPC in the treatment group). These results indicate no drug-specific improvement in the AC/FC of *Fmr1* KO mice. In summary, 1- or 10-day CTEP + minocycline treatment restored gamma power in resting EEG of *Fmr1* KO mice to WT levels but had no major effect on sound-evoked EEG phenotypes in the *Fmr1* KO mice.

### Additional Group Comparisons

Supplemental figures 2–10 show additional comparisons across groups and treatments outside of the three main types of comparisons established a priori at study design. Supplemental Figure 2 shows the effects of vehicle treatment on EEG phenotypes on day 10 of treatment. The phenotypes are less pronounced in the AC and are absent in the FC, suggesting there may be cortical region-specific changes in EEG responses due to the procedure itself. Nevertheless, as shown in Figures 5 and 6, the combined treatment outperformed vehicle in the *Fmr1* KO mice in terms of reducing resting gamma power, indicating a drug effect. Nonetheless, the above data demonstrate and support the benefits of combined drug administration in altering the KO phenotype to reflect WT, especially in the AC. When other types of comparisons are made from 1-day and 10-day post treatment onset (Supplemental Figures 3–10), the procedure itself does not impact the FC gamma band phenotype. Thus, while the primary planned comparisons show that CTEP + minocycline performs better than vehicle in reducing gamma power in KO mice, it should be noted that the FC may be impacted to at least some extent by repeated treatment and handling protocols. This is of importance in designing pre-clinical and clinical studies in the future.

## Discussion

The main goal of this study was to test a combination of drugs that targets two major cellular pathways affected in FXS: mGluR5 and MMP-9. We used CTEP as an antagonist of mGluR5 activity either alone or in combination with an MMP-9 inhibitor, minocycline, in adult male littermate *Fmr1* KO and WT mice to test whether EEG phenotypes are rescued in the *Fmr1* KO mice. We recorded EEG at two time points after treatment, which we refer to as acute (1-day post treatment onset) and chronic (10-day post treatment onset). The major findings can be summarized as: 1) Previously-reported EEG phenotypes in *Fmr1* KO mice tested in non-littermates are present in littermates. Specifically, resting gamma power and STP are elevated in the AC and FC. Additionally, there is a deficit in ITPC of the ASSR in the FC, but not in the AC, as previously reported in non-littermate comparisons of *Fmr1* KO mouse models of FXS (Lovelace et al., 2020). ITPC deficits are also present in the chirp response, but the frequency bands affected in this study are different than previously reported in non-littermate studies (ITPC to 5–30 Hz and 70–80 Hz is significantly reduced and increased, respectively, in this study; vs. ITPC to 10–50 Hz and 60–100 Hz is significantly reduced in Lovelace et al., 2018). 2) CTEP treatment by itself showed no improvement in the EEG phenotype compared to vehicle for either 1-day or 10-day post treatment onset, and in some instances (e.g., STP, Figure 4), CTEP resulted in an exaggerated phenotype compared to vehicle. 3) CTEP + minocycline treatment restored gamma power levels to WT levels and was significantly different from vehicle, on both 1-day and 10-day post treatment onset. There were no effects on sound-evoked responses. The summary of these findings is presented in Table 2, and it suggests a promising approach of combining CTEP and minocycline in treating FXS, with resting EEG power and correlated clinical measures as outcomes.

**Table 2.** Summary of EEG alterations in *Fmr1* KO mice treated with CTEP (gray) or CTEP + minocycline (blue) at day 1 and day 10 post treatment onset.

Treatment	Time points	Resting	STP	ASSR	Chirp
CTEP	DAY 1	1. ✔ Reduced gamma power in the FC in KO group 2. **X** Not different from vehicle		1. ✔ Improved ITPC in the AC and FC in KO group 2. **X** Not different from vehicle	
	DAY 10	1. ✔ Reduced gamma power in the FC in KO group 2. **X** Elevated gamma power compared to vehicle	1. **X** Elevated power in the KO across all frequencies 2. **X** Not different from vehicle in FC, worse than vehicle in the AC	1. ✔ Improved ITPC in the FC in KO group 2. **X** Not different from vehicle	1. ✔ Improved ITPC in the FC in KO group 2. **X** Not different from vehicle in FC, worse than vehicle in the AC
CTEP + mino	DAY 1	1. ✔ Improved gamma power in the AC and FC in KO group 2. ✔ Improved compared to vehicle		1. ✔ Improved ITPC in the AC and FC in KO group 2. **X** Not different from vehicle	
	DAY 10	1. ✔ Improved gamma power in the AC and FC in KO group 2. ✔ Improved compared to vehicle	1. **X** Not improved in gamma frequencies in KO group 2. **X** Not different from vehicle in AC, worse than vehicle in the FC	1. ✔ Improved ITPC in the FC in KO group, **X** made worse in AC 2. **X** Not different from vehicle in FC	1. ✔ Improved ITPC in the AC and FC in KO group 2. **X** Not different from vehicle

Comparisons of treated *Fmr1* KO mice with WT or vehicle-treated *Fmr1* KO mice show (1) drug but not therapeutic effects of CTEP on resting EEG and sound-evoked ITPC deficits, (2) drug and therapeutic effect of CTEP + minocycline on resting EEG deficits, and (3) drug but not therapeutic effects of CTEP + minocycline on sound-evoked potential or ITPC. It is important to note that EEGs from frontal cortex (supplemental figure 2) may show procedure effect under specific comparisons (supplemental figures 2–10). Nevertheless, the combined treatment outperformed vehicle in KO mice in terms of reducing gamma power at rest. CTEP treatment: CTEP dose was 2 mg/kg every 48 hrs. CTEP + minocycline treatment: CTEP dose was 1 mg/kg every 48 hrs, minocycline dose was 15 mg/kg every 24 hrs. STP: single trial power; ASSR: auditory steady state responses.

### Mouse EEG Recordings Reveal Robust, Replicable and Translation-Relevant Phenotypes

In previous studies in *Fmr1* KO mice and in humans with FXS, similar EEG phenotypes have been reported compared to control counterparts. These phenotypes are seen across strains of mice, in the rat model of FXS, and in *in vitro* slice preparations, suggesting a highly reliable and translation-relevant set of outcome measures to test the impact of drug treatments across species (reviewed in Razak et al., 2021). The phenotypes reported in mice and humans include: (1) increased gamma power in resting EEG (Jonak et al., 2020; Lovelace et al., 2018; Wang et al., 2017); (2) reduced phase-locking to spectrotemporally-modulated sound signals such as chirps (Ethridge et al., 2019; Jonak et al., 2020; Lovelace et al., 2018); and (3) increased amplitude of ERPs and STP to auditory stimuli (Ethridge et al., 2017; Knoth & Lippé, 2012; Van der Molen et al., 2012; Wen et al., 2019). The previous epidural mouse work utilized litters of homozygous *Fmr1* KO and WT breeding pairs. However, given that FXS is a neurodevelopmental disorder and that having affected siblings and carrier Het mothers may influence development of WT pups, it was initially crucial to this study to replicate EEG phenotypes in littermate WT/*Fmr1* KO mice. Indeed, the pre-treatment results from the current study show similar EEG phenotypes as previously reported in mice (Figure 2; Lovelace et al., 2018). Changes in other spectral bands are less consistent, including the changes to theta and beta as reported in Lovelace et al. (2020). In the present study, we did not observe any changes in the pre-treatment condition in spectral bands other than gamma.

The use of littermates better isolates the genetic impact of the loss of FMRP expression on EEG measures compared to non-littermate comparisons. Although the frequencies in the chirp stimulus that are impacted in the littermate comparisons (AC, decreased ITPC at ∼10–30 Hz and increased ITPC at ∼70 Hz; FC, decreased ITPC at ∼15 Hz and ∼70–100 Hz) are different from non-littermate comparisons (AC, decreased ITPC at ∼13–50 Hz and ∼70–100 Hz; FC, decreased ITPC at ∼30–100 Hz), the deficit in ITPC to amplitude-modulated signals is persistent across experimental design. Across species, these data show that cortical function in FXS is likely impacted by a milieu of increased total power (elevated STP), variability in response timing across trials (reduced ITPC), reduced habituation to repeated stimuli, and greater synchrony of population responses (larger ERPs) due to hyper-excitability of cortical responses (Rotschafer & Razak, 2013; Wen et al., 2019). These phenotypes will impact auditory processing and lead to high anxiety, abnormal speech and language function, and disrupted social communication, which are hallmark symptoms of FXS and other ASD (Ethridge et al., 2019; Smith et al., 2021; Wilkinson & Nelson, 2021). Similar deficits are also seen in the FC, which will impact hyperactivity and top-down executive function in a task-dependent manner (Sullivan & Brake, 2003).

### CTEP Alone Does Not Affect Evoked or Resting EEG

EEG phenotypes in *Fmr1* KO mice were not improved by CTEP administration alone compared to vehicle at the doses used. This is unlikely to be due to tachyphylaxis (Stoppel et al., 2021) as we did not find any effects after 1 or 10 days on EEG outcomes. The lack of effect of CTEP alone on auditory evoked responses was surprising as mGluR5 is expressed in much of the auditory forebrain and in some parts of the midbrain and brainstem, including the auditory cortex, inferior colliculus, and medial nucleus of the trapezoid body (MNTB; Shigemoto et al., 1997; Romano et al., 1995; Lu, 2014; Curry et al., 2018). mGluR5 plays an important role in auditory processing, including modulation of spontaneous and evoked synaptic inhibition in the brainstem nuclei involved in temporal processing (Curry et al., 2018; Forsythe, 1994). Furthermore, in the AC, presynaptic and postsynaptic group 1 mGluRs regulate excitation and inhibition in cross-regional and cross-laminar circuits (Lu, 2014), possibly resulting in hyper- or hypoexcitable cortical responses to auditory signals. *Fmr1* KO mice exhibit a translationally-upregulated mGluR5 pathway. Previous studies demonstrated that administration of MPEP, an mGluR5 antagonist, in *Fmr1* KO mice rescued a range of behavioral symptoms associated with FXS, including audiogenic seizures in developing mice (AGS; Yan et al., 2005; de Vrij et al., 2008; Gandhi et al., 2014). Furthermore, blocking mGluR5 in the *Fmr1* KO mice rescued neuronal signaling and spine morphology in the hippocampus (de Vrij et al., 2008). Therefore, there was a strong justification to expect that treatment with CTEP, also an mGluR5 antagonist, will modulate auditory responses, particularly those involving phase-locking to temporally modulated stimuli in mice. However, surprisingly, there was either no change in EEG phenotype in the AC or FC of *Fmr1* KO mice following CTEP administration or the phenotype was exaggerated (Figure 4E,F,M,4O: STP and chirp).

The absence of any improvement or, in some cases, the exacerbation of EEG phenotypes with CTEP treatment may be due to the impact of CTEP on mGluR5 activity in parvalbumin-positive (PV) inhibitory neurons. The differences present in the EEG of WT and *Fmr1* KO mice are mostly in the gamma band, with increased resting gamma power, increased STP, and decreased ITPC for 40 Hz ASSR and chirp characterizing the KO phenotype. Altered function of PV inhibitory interneurons will cause deficits in both gamma narrowband oscillations as well as broadband gamma power (Buzsáki & Wang, 2012; Cardin et al., 2009; Chen et al., 2017; Veit et al., 2017). Downregulation of mGluR5 function in PV neurons results in reduced inhibition in hippocampal circuits as demonstrated by fewer mini inhibitory post-synaptic currents in CA1 pyramidal neurons (Barnes et al., 2015). The auditory ERP of mice with PV neuron-specific mGluR5 deletion displayed significantly increased amplitudes in the 10–30 ms and 200 ms post-stimulus window and reduced amplitudes in the 20–60 ms window (Barnes et al., 2015). Notably, stimulus-evoked power was increased in the gamma band, as well as the delta, theta, and beta bands in the frontal and parietal brain regions. Furthermore, autistic-like behaviors were present in these mice, including repetitive behaviors and increased pre-pulse inhibition, reflecting a disruption in sensorimotor gating (Barnes et al., 2015). Billingslea et al. (2014) showed that mGluR5 inhibition via MPEP resulted in worse outcomes in self-care, sociability behaviors, and auditory N1 evoked response latency in mice with PV neuron hypofunction due to loss of NMDA receptors. They suggest that MPEP in combination with increasing PV cell function may prove beneficial. MMP-9 inhibition increases perineuronal net (PNN) expression in PV neurons (Wen et al., 2018). Therefore, reduced gamma power seen with the combined CTEP + minocycline treatment may arise due to reduced mGluR5 activity in conjunction with improved PV cell function, as suggested by Billingslea et al. (2014). Future studies should test the hypothesis that targeting mGluR5 using CTEP or MPEP in combination with a positive allosteric modulator of PV neuron-specific ion channels to improve PV neuron function (Kourdougli et al., 2023) will improve a broad range of EEG phenotypes in *Fmr1* KO mice.

It is important to note that the differential effects of treatment (CTEP alone vs. in combination with minocycline) may be due to dose-dependent effects (2 mg/kg CTEP vs. 1 mg/kg CTEP in the combined treatment), for dose-dependent effects of MTEP, another mGluR5 antagonist, were identified in the primate model for FXS on working memory performance (Yang et al., 2021). Therefore, if this phenomenon is likewise present in the effect of CTEP on the mGluR5 pathway, it is possible that the CTEP dose we used in the CTEP-alone treatment is too high to produce positive results, whereas the lower dose used in the combined treatment has greater efficacy, but this would have to be further tested.

### Unique Effects of Combined Treatment Compared to CTEP or Minocycline Alone

Minocycline administration alone in the *Fmr1* KO group restored phase-locking to temporally modulated signals, namely chirp and ASSR, and reduced sound-induced gamma power (Lovelace et al., 2020). There was no effect on resting EEG power with minocycline treatment. However, in clinical trials, minocycline treatment alone did not result in any EEG-related or behavioral improvement of FXS phenotypes (McKinney et al., 2025). These previous findings led us to investigate the combination of CTEP and minocycline as a potential treatment of established resting EEG and biomarkers for auditory hypersensitivity in FXS. The combination of CTEP and minocycline did not affect sound-evoked responses compared to vehicle, but it significantly reduced resting EEG gamma band power. When combined, there is both an acute (day 1) effect and a chronic effect (day 10) of reduced resting EEG gamma power in the KO mice that is not a placebo effect. This appears to be a unique result of the combination treatment that is not impacted by multiple days of treatment with tachyphylaxis. Stoppel et al. (2021) showed rapid tachyphylaxis to CTEP in visual cortex layer V spontaneous activity in adult mice. This tolerance build-up occurs in the biochemical pathway downstream of GSK3α and upstream of protein synthesis, with chronic reduction of mGluR5 signaling. How minocycline influences biochemical pathways downstream of mGluR5 signaling is unclear. Minocycline carries out multiple functions, including microglia clearance, apoptosis, and inhibition of MMP-9, an endopeptidase that interacts with the extracellular matrix. MMP-9 is elevated in FXS, providing a rationale to test minocycline and other more specific inhibitors of MMP-9. Minocycline treatment reverses mGluR5-dependent hippocampal long-term depression deficits in Fmr1 KO mice (Sidhu et al., 2014). mGluR5 activity promotes downstream production of MMP-9 in the brain (Gkogkas et al., 2014), which is elevated in FXS. Elevated MMP-9 levels in FXS result in increased cleavage of PNNs and may underlie hyperexcitability (Lovelace et al., 2016; Wen et al., 2018). This interaction between mGluR5 and MMP-9 signaling (Gkogkas et al., 2014) may possibly be targeted in a unique manner by the combined treatment, as opposed to minocycline or CTEP alone. It is also possible that these protein cascade interactions reduce tolerance to CTEP in the combined treatment. Future studies should examine the biochemical influence of MMP-9 inhibition on pathways downstream of GSK3α during chronic CTEP administration. Indeed, the clinical efficacy of mGluR5 antagonists may be improved with additional molecules that enhance the benefits of reduced mGluR5 signaling while hindering possible unwanted effects (Dansie et al., 2013). Dual treatment may be the optimal way to target FXS deficits and reverse phenotypes, as it allows for the rescue of multiple categories of symptoms and promotes a more-encompassing treatment strategy. An example of this was demonstrated by Chadwick et al. (2024), in which gaboxadol (GABA receptor agonist) and ibudilast (phosphodiesterase inhibitor) were combined in treatment and improved multiple behavioral phenotypes in FXS.

## Conclusion

This study is one of the first to use cortical EEG measures to assess CTEP treatment effects in *Fmr1* KO animal models of FXS. EEG phenotypes are objective and translatable biomarkers across species. We argue that EEG is a reliable and objective measure for drug effectiveness that offers an intermediate level of analyses between molecular signaling and behavior. We suggest that it is crucial for a drug to demonstrate engagement with FXS-related EEG biomarkers in rodent models before being taken to clinical tests. Specifically, measures of gamma power, temporal processing in response to amplitude-modulated sound signals, and STP to noise burst presentations are objectively similar phenotypes in humans and mice. It is also important to identify EEG-based variability across patients to determine specific outcome measures that might be engaged by a particular drug in a trial. Future studies should also be designed to probe the functional significance of specific EEG outcomes by combining behavioral tasks with EEG recordings.

The benefits of dual-drug treatment on FXS have been previously demonstrated (Chadwick et al., 2024). We found that a combined treatment of CTEP and minocycline yields beneficial effects in the *Fmr1* KO mouse. Specifically, elevated gamma power present in FXS is reduced as a result of combining CTEP and minocycline administration. These benefits are absent with CTEP alone (Figures 3 and 4) or with minocycline treatment alone (Lovelace et al., 2020). Minocycline rescues sound-evoked deficits in *Fmr1* KO mice but not resting phenotype, although this may be due to the increased dose (30 mg/kg) compared to the current study (15 mg/kg). It is important for future preclinical studies to use different dose combinations and routes of administration (i.p. vs. oral gavage) to determine the parameters of optimal treatment. We recommend future clinical treatments to target multiple biomechanistic pathways altered by FXS in combination with EEG outcomes to produce an optimal targeting strategy for treating FXS.

## Data Availability

The data that support the findings of this study are available from the corresponding author [KAR] upon reasonable request.

## References

[CIT0003] Barnes, S. A., Pinto-Duarte, A., Kappe, A., Zembrzycki, A., Metzler, A., Mukamel, E. A., Lucero, J., Wang, X., Sejnowski, T. J., Markou, A., & Behrens, M. M. (2015). Disruption of mGluR5 in parvalbumin-positive interneurons induces core features of neurodevelopmental disorders. *Molecular Psychiatry*, *20*(10), 1161–1172. 10.1038/mp.2015.113 26260494 PMC4583365

[CIT0005] Berry-Kravis, E. M., Harnett, M. D., Reines, S. A., Reese, M. A., Ethridge, L. E., Outterson, A. H., Michalak, C., Furman, J., & Gurney, M. E. (2021). Inhibition of phosphodiesterase-4D in adults with Fragile X Syndrome: A randomized, placebo-controlled, phase 2 clinical trial. *Nature Medicine*, *27*(5), 862–870. 10.1038/s41591-021-01321-w 33927413

[CIT0006] Berry-Kravis, E. M., Lindemann, L., Jønch, A. E., Apostol, G., Bear, M. F., Carpenter, R. L., Crawley, J. N., Curie, A., Des Portes, V., Hossain, F., Gasparini, F., Gomez-Mancilla, B., Hessl, D., Loth, E., Scharf, S. H., Wang, P. P., Von Raison, F., Hagerman, R., Spooren, W., & Jacquemont, S., (2018). Drug development for neurodevelopmental disorders: Lessons learned from Fragile X Syndrome. *Nature Reviews. Drug Discovery*, *17*(4), 280–299. 10.1038/nrd.2017.221 29217836 PMC6904225

[CIT0007] Berry-Kravis, E., Hicar, M., & Ciurlionis, R. (1995). Reduced cyclic AMP production in Fragile X Syndrome: Cytogenetic and molecular correlations. *Pediatric Research*, *38*(5), 638–643. 10.1203/00006450-199511000-00002 8552427

[CIT0008] Bhakar, A. L., Dölen, G., & Bear, M. F. (2012). The pathophysiology of fragile X (and what it teaches us about synapses). *Annual Review of Neuroscience*, *35*(1), 417–443. 10.1146/annurev-neuro-060909-153138 PMC4327822 22483044

[CIT0009] Bhaskaran, A. A., Gauvrit, T., Vyas, Y., Bony, G., Ginger, M., & Frick, A. (2023). Endogenous noise of neocortical neurons correlates with atypical sensory response variability in the Fmr1−/y mouse model of autism. *Nature Communications*, *14*(1), 7905. 10.1038/s41467-023-43777-z PMC10689491 38036566

[CIT0010] Billingslea, E. N., Tatard-Leitman, V. M., Anguiano, J., Jutzeler, C. R., Suh, J., Saunders, J. A., Morita, S., Featherstone, R. E., Ortinski, P. I., Gandal, M. J., Lin, R., Liang, Y., Gur, R. E., Carlson, G. C., Hahn, C.-G., & Siegel, S. J. (2014). Parvalbumin cell ablation of NMDA-R1 causes increased resting network excitability with associated social and self-care deficits. *Neuropsychopharmacology: Official Publication of the American College of Neuropsychopharmacology*, *39*(7), 1603–1613. 10.1038/npp.2014.7 24525709 PMC4023157

[CIT0011] Bilousova, T. V., Dansie, L., Ngo, M., Aye, J., Charles, J. R., Ethell, D. W., & Ethell, I. M. (2009). Minocycline promotes dendritic spine maturation and improves behavioural performance in the fragile X mouse model. *Journal of Medical Genetics*, *46*(2), 94–102. 10.1136/jmg.2008.061796 18835858

[CIT0012] Budimirovic, D. B., Dominick, K. C., Gabis, L. V., Adams, M., Adera, M., Huang, L., Ventola, P., Tartaglia, N. R., & Berry-Kravis, E. (2021). Gaboxadol in Fragile X Syndrome: A 12-week randomized, double-blind, parallel-group, phase 2a study. *Frontiers in Pharmacology*, *12*, 757825. 10.3389/fphar.2021.757825 34690787 PMC8531725

[CIT0013] Buzsáki, G., & Wang, X.-J. (2012). Mechanisms of gamma oscillations. *Annual Review of Neuroscience*, *35*(1), 203–225. 10.1146/annurev-neuro-062111-150444 PMC4049541 22443509

[CIT0014] Cardin, J. A., Carlén, M., Meletis, K., Knoblich, U., Zhang, F., Deisseroth, K., Tsai, L.-H., & Moore, C. I. (2009). Driving fast-spiking cells induces gamma rhythm and controls sensory responses. *Nature*, *459*(7247), 663–667. 10.1038/nature08002 19396156 PMC3655711

[CIT0015] Chadwick, W., Angulo-Herrera, I., Cogram, P., Deacon, R. J. M., Mason, D. J., Brown, D., Roberts, I., O’Donovan, D. J., Tranfaglia, M. R., Guilliams, T., & Thompson, N. T. (2024). A novel combination treatment for Fragile X Syndrome predicted using computational methods. *Brain Communications*, *6*(1), fcad353. 10.1093/braincomms/fcad353 38226317 PMC10789243

[CIT0016] Champigny, C., Morin-Parent, F., Bellehumeur-Lefebvre, L., Çaku, A., Lepage, J. F., & Corbin, F. (2021). Combining lovastatin and minocycline for the treatment of Fragile X Syndrome: Results from the LovaMiX clinical trial. *Frontiers in Psychiatry*, *12*, 762967. 10.3389/fpsyt.2021.762967 35058813 PMC8763805

[CIT0017] Chen, G., Zhang, Y., Li, X., Zhao, X., Ye, Q., Lin, Y., Tao, H. W., Rasch, M. J., & Zhang, X. (2017). Distinct inhibitory circuits orchestrate cortical beta and gamma band oscillations. *Neuron*, *96*(6), 1403–1418.e6. 10.1016/j.neuron.2017.11.033 29268099 PMC5864125

[CIT0018] Chuang, S. C., Zhao, W., Bauchwitz, R., Yan, Q., Bianchi, R., & Wong, R. K. (2005). Prolonged epileptiform discharges induced by altered group I metabotropic glutamate receptor-mediated synaptic responses in hippocampal slices of a fragile X mouse model. *The Journal of Neuroscience: The Official Journal of the Society for Neuroscience*, *25*(35), 8048–8055. 10.1523/JNEUROSCI.1777-05.2005 16135762 PMC6725444

[CIT0019] Crawford, D. C., Acuña, J. M., & Sherman, S. L. (2001). FMR1 and the Fragile X Syndrome: Human genome epidemiology review. *Genetics in Medicine: Official Journal of the American College of Medical Genetics*, *3*(5), 359–371. 10.1097/00125817-200109000-00006 11545690 PMC4493892

[CIT0020] Croom, K., Rumschlag, J. A., Erickson, M. A., Binder, D. K., & Razak, K. A. (2023). Developmental delays in cortical auditory temporal processing in a mouse model of Fragile X Syndrome. *Journal of Neurodevelopmental Disorders*, *15*(1), 23. 10.1186/s11689-023-09496-8 37516865 PMC10386252

[CIT0021] Curry, R. J., Peng, K., & Lu, Y. (2018). Neurotransmitter-and release-mode-specific modulation of inhibitory transmission by group I metabotropic glutamate receptors in central auditory neurons of the mouse. *The Journal of Neuroscience: The Official Journal of the Society for Neuroscience*, *38*(38), 8187–8199. 10.1523/JNEUROSCI.0603-18.2018 30093538 PMC6146499

[CIT0022] Dansie, L. E., Phommahaxay, K., Okusanya, A. G., Uwadia, J., Huang, M., Rotschafer, S. E., Razak, K. A., Ethell, D. W., & Ethell, I. M. (2013). Long-lasting effects of minocycline on behavior in young but not adult Fragile X mice. *Neuroscience*, *246*, 186–198. 10.1016/j.neuroscience.2013.04.058 23660195 PMC3813005

[CIT0023] de Vrij, F. M., Levenga, J., Van der Linde, H. C., Koekkoek, S. K., De Zeeuw, C. I., Nelson, D. L., Oostra, B. A., & Willemsen, R. (2008). Rescue of behavioral phenotype and neuronal protrusion morphology in Fmr1 KO mice. *Neurobiology of Disease*, *31*(1), 127–132. 10.1016/j.nbd.2008.04.002 18571098 PMC2481236

[CIT0024] Dölen, G., & Bear, M. F. (2008). Role for metabotropic glutamate receptor 5 (mGluR5) in the pathogenesis of Fragile X Syndrome. *The Journal of Physiology*, *586*(6), 1503–1508. 10.1113/jphysiol.2008.150722 18202092 PMC2375688

[CIT0025] Dölen, G., Osterweil, E., Rao, B. S., Smith, G. B., Auerbach, B. D., Chattarji, S., & Bear, M. F. (2007). Correction of Fragile X Syndrome in mice. *Neuron*, *56*(6), 955–962. 10.1016/j.neuron.2007.12.001 18093519 PMC2199268

[CIT0026] Dziembowska, M. (2023). How dendritic spines shape is determined by MMP-9 activity in FXS. *International Review of Neurobiology*, *173*, 171–185. 10.1016/bs.irn.2023.10.001 37993177

[CIT0027] Dziembowska, M., Pretto, D. I., Janusz, A., Kaczmarek, L., Leigh, M. J., Gabriel, N., Durbin‐Johnson, B., Hagerman, R. J., & Tassone, F. (2013). High MMP‐9 activity levels in Fragile X Syndrome are lowered by minocycline. *American Journal of Medical Genetics. Part A*, *161A*(8), 1897–1903. 10.1002/ajmg.a.36023 23824974

[CIT0028] Erickson, C. A., Shaffer, R. C., Will, M., Schmitt, L. M., Horn, P., Hirst, K., Pedapati, E. V., Ober, N., Tumuluru, R. V., Handen, B. L., & Beversdorf, D. Q. (2025). Brief report: A double-blind, placebo-controlled, crossover, proof-of-concept study of minocycline in autism spectrum disorder. *Journal of Autism and Developmental Disorders*, *55*(9), 3387–3394. 10.1007/s10803-023-06132-1 38102393

[CIT0029] Ethell, I. M., & Ethell, D. W. (2007). Matrix metalloproteinases in brain development and remodeling: Synaptic functions and targets. *Journal of Neuroscience Research*, *85*(13), 2813–2823. 10.1002/jnr.21273 17387691

[CIT0030] Ethridge, L. E., De Stefano, L. A., Schmitt, L. M., Woodruff, N. E., Brown, K. L., Tran, M., Wang, J., Pedapati, E. V., Erickson, C. A., & Sweeney, J. A. (2019). Auditory EEG biomarkers in Fragile X Syndrome: Clinical relevance. *Frontiers in Integrative Neuroscience*, *13*, 60. 10.3389/fnint.2019.00060 31649514 PMC6794497

[CIT0031] Ethridge, L. E., White, S. P., Mosconi, M. W., Wang, J., Pedapati, E. V., Erickson, C. A., Byerly, M. J., & Sweeney, J. A. (2017). Neural synchronization deficits linked to cortical hyper-excitability and auditory hypersensitivity in Fragile X Syndrome. *Molecular Autism*, *8*, 38. 10.1186/s13229-017-0140-1 28596820 PMC5463459

[CIT0032] Forsythe, I. D. (1994). Direct patch recording from identified presynaptic terminals mediating glutamatergic EPSCs in the rat CNS, in vitro. *The Journal of Physiology*, *479*(Pt 3), 381–387. 10.1113/jphysiol.1994.sp020303 7837096 PMC1155757

[CIT0033] Gandhi, R. M., Kogan, C. S., & Messier, C. (2014). 2-Methyl-6-(phenylethynyl) pyridine (MPEP) reverses maze learning and PSD-95 deficits in Fmr1 knock-out mice. *Frontiers in Cellular Neuroscience*, *8*, 70. 10.3389/fncel.2014.00070 24701200 PMC3965849

[CIT0034] Gkogkas, C. G., Khoutorsky, A., Cao, R., Jafarnejad, S. M., Prager-Khoutorsky, M., Giannakas, N., Kaminari, A., Fragkouli, A., Nader, K., Price, T. J., Konicek, B. W., Graff, J. R., Tzinia, A. K., Lacaille, J.-C., & Sonenberg, N. (2014). Pharmacogenetic inhibition of eIF4E-dependent Mmp9 mRNA translation reverses Fragile X Syndrom -like phenotypes. *Cell Reports*, *9*(5), 1742–1755. 10.1016/j.celrep.2014.10.064 25466251 PMC4294557

[CIT0035] Gore, S. V., James, E. J., Huang, L. C., Park, J. J., Berghella, A., Thompson, A. C., Cline, H. T., & Aizenman, C. D. (2021). Role of matrix metalloproteinase-9 in neurodevelopmental deficits and experience-dependent plasticity in Xenopus laevis. *eLife*, *10*, e62147. 10.7554/eLife.62147 34282726 PMC8315794

[CIT0036] Hamilton, A., Vasefi, M., Vander Tuin, C., McQuaid, R. J., Anisman, H., & Ferguson, S. S. (2016). Chronic pharmacological mGluR5 inhibition prevents cognitive impairment and reduces pathogenesis in an Alzheimer disease mouse model. *Cell Reports*, *15*(9), 1859–1865. 10.1016/j.celrep.2016.04.077 27210751

[CIT0037] Huber, K. M., Gallagher, S. M., Warren, S. T., & Bear, M. F. (2002). Altered synaptic plasticity in a mouse model of fragile X mental retardation. *Proceedings of the National Academy of Sciences of the United States of America*, *99*(11), 7746–7750. 10.1073/pnas.122205699 12032354 PMC124340

[CIT0038] Janusz, A., Milek, J., Perycz, M., Pacini, L., Bagni, C., Kaczmarek, L., & Dziembowska, M. (2013). The Fragile X mental retardation protein regulates matrix metalloproteinase 9 mRNA at synapses. *The Journal of Neuroscience: The Official Journal of the Society for Neuroscience*, *33*(46), 18234–18241. 10.1523/JNEUROSCI.2207-13.2013 24227732 PMC6619756

[CIT0039] Janz, P., Bainier, M., Marashli, S., Gross, S., & Redondo, R. L. (2025). Clinically-probed mechanisms of action in Fragile-X Syndrome fail to normalize translational EEG phenotypes in Fmr1 knockout mice. *Neuropharmacology*, *262*, 110182. 10.1016/j.neuropharm.2024.110182 39396738

[CIT0040] Jin, P., & Warren, S. T. (2000). Understanding the molecular basis of Fragile X Syndrome. *Human Molecular Genetics*, *9*(6), 901–908. 10.1093/hmg/9.6.901 10767313

[CIT0041] Jonak, C. R., Assad, S. A., Garcia, T. A., Sandhu, M. S., Rumschlag, J. A., Razak, K. A., & Binder, D. K. (2024). Phenotypic analysis of multielectrode array EEG biomarkers in developing and adult male Fmr1 KO mice. *Neurobiology of Disease*, *195*, 106496. 10.1016/j.nbd.2024.106496 38582333

[CIT0042] Jonak, C. R., Lovelace, J. W., Ethell, I. M., Razak, K. A., & Binder, D. K. (2018). Reusable multielectrode array technique for electroencephalography in awake freely moving mice. *Frontiers in Integrative Neuroscience*, *12*, 53. 10.3389/fnint.2018.00053 30416434 PMC6213968

[CIT0043] Jonak, C. R., Lovelace, J. W., Ethell, I. M., Razak, K. A., & Binder, D. K. (2020). Multielectrode array analysis of EEG biomarkers in a mouse model of Fragile X Syndrome. *Neurobiology of Disease*, *138*, 104794. 10.1016/j.nbd.2020.104794 32036032 PMC9038039

[CIT0044] Jonak, C. R., Sandhu, M. S., Assad, S. A., Barbosa, J. A., Makhija, M., & Binder, D. K. (2021). The PDE10A inhibitor TAK-063 reverses sound-evoked EEG abnormalities in a mouse model of Fragile X Syndrome. *Neurotherapeutics: The Journal of the American Society for Experimental NeuroTherapeutics*, *18*(2), 1175–1187. 10.1007/s13311-021-01005-w 33594533 PMC8423959

[CIT0045] Kanellopoulos, A. K., Semelidou, O., Kotini, A. G., Anezaki, M., & Skoulakis, E. M. (2012). Learning and memory deficits consequent to reduction of the fragile X mental retardation protein result from metabotropic glutamate receptor-mediated inhibition of cAMP signaling in Drosophila. *The Journal of Neuroscience: The Official Journal of the Society for Neuroscience*, *32*(38), 13111–13124. 10.1523/JNEUROSCI.1347-12.2012 22993428 PMC6621471

[CIT0046] Knoth, I. S., & Lippé, S. (2012). Event-related potential alterations in Fragile X Syndrome. *Frontiers in Human Neuroscience*, *6*, 264. 10.3389/fnhum.2012.00264 23015788 PMC3449440

[CIT0047] Kohl, C., Parviainen, T., & Jones, S. R. (2022). Neural mechanisms underlying human auditory evoked responses revealed by human neocortical neurosolver. *Brain Topography*, *35*(1), 19–35. 10.1007/s10548-021-00838-0 33876329 PMC8813713

[CIT0048] Kokash, J., Alderson, E. M., Reinhard, S. M., Crawford, C. A., Binder, D. K., Ethell, I. M., & Razak, K. A. (2019). Genetic reduction of MMP-9 in the Fmr1 KO mouse partially rescues prepulse inhibition of acoustic startle response. *Brain Research*, *1719*, 24–29. 10.1016/j.brainres.2019.05.029 31128097 PMC6640842

[CIT0049] Kourdougli, N., Suresh, A., Liu, B., Juarez, P., Lin, A., Chung, D. T., Graven Sams, A., Gandal, M. J., Martínez-Cerdeño, V., Buonomano, D. V., Hall, B. J., Mombereau, C., & Portera-Cailliau, C., (2023). Improvement of sensory deficits in fragile X mice by increasing cortical interneuron activity after the critical period. *Neuron*, *111*(18), 2863–2880.e6. 10.1016/j.neuron.2023.06.009 37451263 PMC10529373

[CIT0050] Kremer, E. J., Pritchard, M., Lynch, M., Yu, S., Holman, K., Baker, E., Warren, S. T., Schlessinger, D., Sutherland, G. R., & Richards, R. I. (1991). Mapping of DNA instability at the fragile X to a trinucleotide repeat sequence p(CCG)n. *Science (New York, N.Y.)*, *252*(5013), 1711–1714. 10.1126/science.1675488 1675488

[CIT0051] Laroui, A., Galarneau, L., Abolghasemi, A., Benachenhou, S., Plantefève, R., Bouchouirab, F. Z., Lepage, J. F., Corbin, F., & Çaku, A. (2022). Clinical significance of matrix metalloproteinase-9 in Fragile X Syndrome. *Scientific Reports*, *12*(1), 15386. 10.1038/s41598-022-19476-y 36100610 PMC9470743

[CIT0052] Lindemann, L., Jaeschke, G., Michalon, A., Vieira, E., Honer, M., Spooren, W., Porter, R., Hartung, T., Kolczewski, S., Büttelmann, B., Flament, C., Diener, C., Fischer, C., Gatti, S., Prinssen, E. P., Parrott, N., Hoffmann, G., & Wettstein, J. G. (2011). CTEP: a novel, potent, long-acting, and orally bioavailable metabotropic glutamate receptor 5 inhibitor. *The Journal of Pharmacology and Experimental Therapeutics*, *339*(2), 474–486. 10.1124/jpet.111.185660 21849627

[CIT0053] Lovelace, J. W., Ethell, I. M., Binder, D. K., & Razak, K. A. (2018). Translation-relevant EEG phenotypes in a mouse model of Fragile X Syndrome. *Neurobiology of Disease*, *115*, 39–48. 10.1016/j.nbd.2018.03.012 29605426 PMC5969806

[CIT0054] Lovelace, J. W., Ethell, I. M., Binder, D. K., & Razak, K. A. (2020). Minocycline treatment reverses sound evoked EEG abnormalities in a mouse model of Fragile X Syndrome. *Frontiers in Neuroscience*, *14*, 771. 10.3389/fnins.2020.00771 32848552 PMC7417521

[CIT0055] Lovelace, J. W., Wen, T. H., Reinhard, S., Hsu, M. S., Sidhu, H., Ethell, I. M., Binder, D. K., & Razak, K. A. (2016). Matrix metalloproteinase-9 deletion rescues auditory evoked potential habituation deficit in a mouse model of Fragile X Syndrome. *Neurobiology of Disease*, *89*, 126–135. 10.1016/j.nbd.2016.02.002 26850918 PMC4785038

[CIT0056] Lu, Y. (2014). Metabotropic glutamate receptors in auditory processing. *Neuroscience*, *274*, 429–445. 10.1016/j.neuroscience.2014.05.057 24909898 PMC5299851

[CIT0057] Luján, R., Roberts, J. D. B., Shigemoto, R., Ohishi, H., & Somogyi, P. (1997). Differential plasma membrane distribution of metabotropic glutamate receptors mGluR1α, mGluR2 and mGluR5, relative to neurotransmitter release sites. *Journal of Chemical Neuroanatomy*, *13*(4), 219–241. 10.1016/s0891-0618(97)00051-3 9412905

[CIT0058] Luu, S., Province, H., Berry-Kravis, E., Hagerman, R., Hessl, D., Vaidya, D., Lozano, R., Rosselot, H., Erickson, C., Kaufmann, W. E., & Budimirovic, D. B. (2020). Response to placebo in Fragile X Syndrome clinical trials: an initial analysis. *Brain Sciences*, *10*(9), 629. 10.3390/brainsci10090629 32932789 PMC7563217

[CIT0059] Magnowska, M., Gorkiewicz, T., Suska, A., Wawrzyniak, M., Rutkowska-Wlodarczyk, I., Kaczmarek, L., & Wlodarczyk, J. (2016). Transient ECM protease activity promotes synaptic plasticity. *Scientific Reports*, *6*(1), 27757. 10.1038/srep27757 27282248 PMC4901294

[CIT0060] Maris, E., & Oostenveld, R. (2007). Nonparametric statistical testing of EEG-and MEG-data. *Journal of Neuroscience Methods*, *164*(1), 177–190. 10.1016/j.jneumeth.2007.03.024 17517438

[CIT0061] McBride, S. M. J., Choi, C. H., Wang, Y., Liebelt, D., Braunstein, E., Ferreiro, D., Sehgal, A., Siwicki, K. K., Dockendorff, T. C., Nguyen, H. T., McDonald, T. V., & Jongens, T. A. (2005). Pharmacological rescue of synaptic plasticity, courtship behavior, and mushroom body defects in a Drosophila model of Fragile X Syndrome. *Neuron*, *45*(5), 753–764. 10.1016/j.neuron.2005.01.038 15748850

[CIT0062] McKinney, W. S., Schmitt, L. M., De Stefano, L. A., Ethridge, L., Norris, J. E., Horn, P. S., Dauterman, S., Rosselot, H., Pedapati, E. V., Reisinger, D. L., Dominick, K. C., Shaffer, R. C., Chin, D., Friedman, N. R., Hong, M., Sweeney, J. A., & Erickson, C. (2025). Results from a double-blind, randomized, placebo-controlled, single-dose, crossover trial of lovastatin or minocycline in Fragile X Syndrome. *Journal of Child and Adolescent Psychopharmacology*, *35*(4), 211–221. 10.1089/cap.2024.0103 39651602 PMC12143942

[CIT0063] Michalon, A., Sidorov, M., Ballard, T. M., Ozmen, L., Spooren, W., Wettstein, J. G., Jaeschke, G., Bear, M. F., & Lindemann, L. (2012). Chronic pharmacological mGlu5 inhibition corrects fragile X in adult mice. *Neuron*, *74*(1), 49–56. 10.1016/j.neuron.2012.03.009 22500629 PMC8822597

[CIT0064] Neymotin, S. A., Daniels, D. S., Caldwell, B., McDougal, R. A., Carnevale, N. T., Jas, M., Moore, C. I., Hines, M. L., Hämäläinen, M., & Jones, S. R. (2020). Human Neocortical Neurosolver (HNN), a new software tool for interpreting the cellular and network origin of human MEG/EEG data. *eLife*, *9*, e51214. 10.7554/eLife.51214 31967544 PMC7018509

[CIT0065] Niswender, C. M., & Conn, P. J. (2010). Metabotropic glutamate receptors: Physiology, pharmacology, and disease. *Annual Review of Pharmacology and Toxicology*, *50*(1), 295–322. 10.1146/annurev.pharmtox.011008.145533 PMC2904507 20055706

[CIT0066] O’Donnell, B. F., Vohs, J. L., Krishnan, G. P., Rass, O., Hetrick, W. P., & Morzorati, S. L. (2013). The auditory steady-state response (ASSR): A translational biomarker for schizophrenia. *Supplements to Clinical Neurophysiology*, *62*, 101–112. 10.1016/b978-0-7020-5307-8.00006-5 24053034 PMC4959266

[CIT0067] Oberlé, I., Rousseau, F., Heitz, D., Kretz, C., Devys, D., Hanauer, A., Boué, J., Bertheas, M. F., & Mandel, J. L. (1991). Instability of a 550-base pair DNA segment and abnormal methylation in Fragile X Syndrome. *Science (New York, N.Y.)*, *252*(5009), 1097–1102. 10.1126/science.252.5009.1097 2031184

[CIT0068] Paluszkiewicz, S. M., Martin, B. S., & Huntsman, M. M. (2011). Fragile X Syndrome: The GABAergic system and circuit dysfunction. *Developmental Neuroscience*, *33*(5), 349–364. 10.1159/000329420 21934270 PMC3254035

[CIT0069] Pirbhoy, P. S., Rais, M., Lovelace, J. W., Woodard, W., Razak, K. A., Binder, D. K., & Ethell, I. M. (2020). Acute pharmacological inhibition of matrix metalloproteinase‐9 activity during development restores perineuronal net formation and normalizes auditory processing in Fmr1 KO mice. *Journal of Neurochemistry*, *155*(5), 538–558. 10.1111/jnc.15037 32374912 PMC7644613

[CIT0070] Purcell, D. W., John, S. M., Schneider, B. A., & Picton, T. W. (2004). Human temporal auditory acuity as assessed by envelope following responses. *The Journal of the Acoustical Society of America*, *116*(6), 3581–3593. 10.1121/1.1798354 15658709

[CIT0071] Rais, M., Binder, D. K., Razak, K. A., & Ethell, I. M. (2018). Sensory processing phenotypes in Fragile X Syndrome. *ASN Neuro*, *10*(1), 1759091418801092. 10.1177/1759091418801092 30231625 PMC6149018

[CIT0072] Rankin‐Gee, E. K., McRae, P. A., Baranov, E., Rogers, S., Wandrey, L., & Porter, B. E. (2015). Perineuronal net degradation in epilepsy. *Epilepsia*, *56*(7), 1124–1133. 10.1111/epi.13026 26032766

[CIT0073] Razak, K. A., Binder, D. K., & Ethell, I. M. (2021). Neural correlates of auditory hypersensitivity in Fragile X Syndrome. *Frontiers in Psychiatry*, *12*, 720752. 10.3389/fpsyt.2021.720752 34690832 PMC8529206

[CIT0074] Reinhard, S. M., Razak, K., & Ethell, I. M. (2015). A delicate balance: Role of MMP-9 in brain development and pathophysiology of neurodevelopmental disorders. *Frontiers in Cellular Neuroscience*, *9*, 280. 10.3389/fncel.2015.00280 26283917 PMC4518323

[CIT0075] Romano, C., Sesma, M. A., McDonald, C. T., O’malley, K., van den Pol, A. N., & Olney, J. W. (1995). Distribution of metabotropic glutamate receptor mGluR5 immunoreactivity in rat brain. *The Journal of Comparative Neurology*, *355*(3), 455–469. 10.1002/cne.903550310 7636025

[CIT0076] Rotschafer, S. E., Trujillo, M. S., Dansie, L. E., Ethell, I. M., & Razak, K. A. (2012). Minocycline treatment reverses ultrasonic vocalization production deficit in a mouse model of Fragile X Syndrome. *Brain Research*, *1439*, 7–14. 10.1016/j.brainres.2011.12.041 22265702

[CIT0077] Rotschafer, S., & Razak, K. (2013). Altered auditory processing in a mouse model of Fragile X Syndrome. *Brain Research*, *1506*, 12–24. 10.1016/j.brainres.2013.02.038 23458504

[CIT0078] Rumschlag, J. A., Lovelace, J. W., & Razak, K. A. (2021). Age-and movement-related modulation of cortical oscillations in a mouse model of presbycusis. *Hearing Research*, *402*, 108095. 10.1016/j.heares.2020.108095 33707000

[CIT0079] Schneider, A., Leigh, M. J., Adams, P., Nanakul, R., Chechi, T., Olichney, J., Hagerman, R., & Hessl, D. (2013). Electrocortical changes associated with minocycline treatment in Fragile X Syndrome. *Journal of Psychopharmacology (Oxford, England)*, *27*(10), 956–963. 10.1177/0269881113494105 23981511 PMC4962861

[CIT0080] Seymour, R. A., Rippon, G., Gooding-Williams, G., Sowman, P. F., & Kessler, K. (2020). Reduced auditory steady state responses in autism spectrum disorder. *Molecular Autism*, *11*(1), 56. 10.1186/s13229-020-00357-y 32611372 PMC7329477

[CIT0081] Shigemoto, R., Kinoshita, A., Wada, E., Nomura, S., Ohishi, H., Takada, M., Flor, P. J., Neki, A., Abe, T., Nakanishi, S., & Mizuno, N. (1997). Differential presynaptic localization of metabotropic glutamate receptor subtypes in the rat hippocampus. *The Journal of Neuroscience: The Official Journal of the Society for Neuroscience*, *17*(19), 7503–7522. 10.1523/JNEUROSCI.17-19-07503.1997 9295396 PMC6573434

[CIT0082] Sidhu, H., Dansie, L. E., Hickmott, P. W., Ethell, D. W., & Ethell, I. M. (2014). Genetic removal of matrix metalloproteinase 9 rescues the symptoms of Fragile X Syndrome in a mouse model. *The Journal of Neuroscience: The Official Journal of the Society for Neuroscience*, *34*(30), 9867–9879. 10.1523/JNEUROSCI.1162-14.2014 25057190 PMC4107404

[CIT0083] Smith, E. G., Pedapati, E. V., Liu, R., Schmitt, L. M., Dominick, K. C., Shaffer, R. C., Sweeney, J. A., & Erickson, C. A. (2021). Sex differences in resting EEG power in Fragile X Syndrome. *Journal of Psychiatric Research*, *138*, 89–95. 10.1016/j.jpsychires.2021.03.057 33836434 PMC8192450

[CIT0084] Stawarski, M., Stefaniuk, M., & Wlodarczyk, J. (2014). Matrix metalloproteinase-9 involvement in the structural plasticity of dendritic spines. *Frontiers in Neuroanatomy*, *8*, 68. 10.3389/fnana.2014.00068 25071472 PMC4091410

[CIT0085] Stoppel, D. C., McCamphill, P. K., Senter, R. K., Heynen, A. J., & Bear, M. F. (2021). mGluR5 Negative modulators for fragile X: Treatment resistance and persistence. *Frontiers in Psychiatry*, *12*, 718953. 10.3389/fpsyt.2021.718953 34658956 PMC8511445

[CIT0086] Stoppel, L. J., Osterweil, E. K., & Bear, M. F. (2017). The mGluR theory of fragile X: From mice to men. In *Fragile X Syndrome* (pp. 173–204). Academic Press.

[CIT0087] Sullivan, R. M., & Brake, W. G. (2003). What the rodent prefrontal cortex can teach us about attention-deficit/hyperactivity disorder: The critical role of early developmental events on prefrontal function. *Behavioural Brain Research*, *146*(1–2), 43–55. 10.1016/j.bbr.2003.09.015 14643458

[CIT0088] Taschenberger, H., & Von Gersdorff, H. (2000). Fine-tuning an auditory synapse for speed and fidelity: Developmental changes in presynaptic waveform, EPSC kinetics, and synaptic plasticity. *The Journal of Neuroscience: The Official Journal of the Society for Neuroscience*, *20*(24), 9162–9173. 10.1523/JNEUROSCI.20-24-09162.2000 11124994 PMC6773022

[CIT0089] Toledo, M. A., Wen, T. H., Binder, D. K., Ethell, I. M., & Razak, K. A. (2019). Reversal of ultrasonic vocalization deficits in a mouse model of Fragile X Syndrome with minocycline treatment or genetic reduction of MMP-9. *Behavioural Brain Research*, *372*, 112068. 10.1016/j.bbr.2019.112068 31271818 PMC6662633

[CIT0090] Toso, A., Wermuth, A. P., Arazi, A., Braun, A., Grent, T., Uhlhaas, P. J., & Donner, T. H. (2024). 40 Hz Steady-state response in human auditory cortex is shaped by gabaergic neuronal inhibition. *Journal of Neuroscience*, (24), 44. 38670804 10.1523/JNEUROSCI.2029-23.2024 PMC11170946

[CIT0091] Tucker, B., Richards, R. I., & Lardelli, M. (2006). Contribution of mGluR and Fmr1 functional pathways to neurite morphogenesis, craniofacial development and Fragile X Syndrome. *Human Molecular Genetics*, *15*(23), 3446–3458. 10.1093/hmg/ddl422 17065172

[CIT0092] Van der Molen, M. J. W., Van der Molen, M. W., Ridderinkhof, K. R., Hamel, B. C. J., Curfs, L. M. G., & Ramakers, G. J. A. (2012). Auditory and visual cortical activity during selective attention in Fragile X Syndrome: A cascade of processing deficiencies. *Clinical Neurophysiology: official Journal of the International Federation of Clinical Neurophysiology*, *123*(4), 720–729. 10.1016/j.clinph.2011.08.023 21958658

[CIT0093] Veit, J., Hakim, R., Jadi, M. P., Sejnowski, T. J., & Adesnik, H. (2017). Cortical gamma band synchronization through somatostatin interneurons. *Nature Neuroscience*, *20*(7), 951–959. 10.1038/nn.4562 28481348 PMC5511041

[CIT0094] Verkerk, A. J., Pieretti, M., Sutcliffe, J. S., Fu, Y. H., Kuhl, D. P., Pizzuti, A., Reiner, O., Richards, S., Victoria, M. F., & Zhang, F. P. (1991). Identification of a gene (FMR-1) containing a CGG repeat coincident with a breakpoint cluster region exhibiting length variation in Fragile X Syndrome. *Cell*, *65*(5), 905–914. 10.1016/0092-8674(91)90397-h 1710175

[CIT0095] Wang, H., Ferguson, G. D., Pineda, V. V., Cundiff, P. E., & Storm, D. R. (2004). Overexpression of type-1 adenylyl cyclase in mouse forebrain enhances recognition memory and LTP. *Nature Neuroscience*, *7*(6), 635–642. 10.1038/nn1248 15133516

[CIT0096] Wang, J., Ethridge, L. E., Mosconi, M. W., White, S. P., Binder, D. K., Pedapati, E. V., Erickson, C. A., Byerly, M. J., & Sweeney, J. A. (2017). A resting EEG study of neocortical hyperexcitability and altered functional connectivity in Fragile X Syndrome. *Journal of Neurodevelopmental Disorders*, *9*(1), 11. 10.1186/s11689-017-9191-z 28316753 PMC5351111

[CIT0097] Warren, S. T., & Nelson, D. L. (1994). Advances in molecular analysis of Fragile X Syndrome. *JAMA: The Journal of the American Medical Association*, *271*(7), 536–542. 10.1001/jama.1994.03510310066040 8301769

[CIT0098] Wen, T. H., Afroz, S., Reinhard, S. M., Palacios, A. R., Tapia, K., Binder, D. K., Razak, K. A., & Ethell, I. M. (2018). Genetic reduction of matrix metalloproteinase-9 promotes formation of perineuronal nets around parvalbumin-expressing interneurons and normalizes auditory cortex responses in developing Fmr1 knock-out mice. *Cerebral Cortex (New York, N.Y.: 1991)*, *28*(11), 3951–3964. 10.1093/cercor/bhx258 29040407 PMC6188540

[CIT0099] Wen, T. H., Lovelace, J. W., Ethell, I. M., Binder, D. K., & Razak, K. A. (2019). Developmental changes in EEG phenotypes in a mouse model of Fragile X Syndrome. *Neuroscience*, *398*, 126–143. 10.1016/j.neuroscience.2018.11.047 30528856 PMC6331246

[CIT0100] Wilkinson, C. L., & Nelson, C. A. (2021). Increased aperiodic gamma power in young boys with Fragile X Syndrome is associated with better language ability. *Molecular Autism*, *12*(1), 17. 10.1186/s13229-021-00425-x 33632320 PMC7908768

[CIT0101] Yan, Q. J., Rammal, M., Tranfaglia, M., & Bauchwitz, R. P. (2005). Suppression of two major Fragile X Syndrome mouse model phenotypes by the mGluR5 antagonist MPEP. *Neuropharmacology*, *49*(7), 1053–1066. 10.1016/j.neuropharm.2005.06.004 16054174

[CIT0102] Yang, S. T., Wang, M., Galvin, V., Yang, Y., & Arnsten, A. F. (2021). Effects of blocking mGluR5 on primate dorsolateral prefrontal cortical neuronal firing and working memory performance. *Psychopharmacology*, *238*(1), 97–106. 10.1007/s00213-020-05661-2 32939596 PMC7794104

[CIT0103] Yu, S., Pritchard, M., Kremer, E., Lynch, M., Nancarrow, J., Baker, E., Holman, K., Mulley, J. C., Warren, S. T., & Schlessinger, D. (1991). Fragile X genotype characterized by an unstable region of DNA. *Science (New York, N.Y.)*, *252*(5009), 1179–1181. 10.1126/science.252.5009.1179 2031189

